# The Effect of Interference on the CD8^+^ T Cell Escape Rates in HIV

**DOI:** 10.3389/fimmu.2014.00661

**Published:** 2015-01-13

**Authors:** Victor Garcia, Roland Robert Regoes

**Affiliations:** ^1^Institute of Integrative Biology, Department of Environmental Systems Science, ETH Zürich, Zurich, Switzerland

**Keywords:** theoretical biology, mathematical modeling, HIV dynamics, interference, cytotoxic T lymphocytes, escape

## Abstract

In early human immunodeficiency virus (HIV) infection, the virus population escapes from multiple CD8^+^ cell responses. The later an escape mutation emerges, the slower it outgrows its competition, i.e., the escape rate is lower. This pattern could indicate that the strength of the CD8^+^ cell responses is waning, or that later viral escape mutants carry a larger fitness cost. In this paper, we investigate whether the pattern of decreasing escape rates could also be caused by genetic interference among different escape strains. To this end, we developed a mathematical multi-epitope model of HIV dynamics, which incorporates stochastic effects, recombination, and mutation. We used cumulative linkage disequilibrium measures to quantify the amount of interference. We found that nearly synchronous, similarly strong immune responses in two-locus systems enhance the generation of genetic interference. This effect, combined with a scheme of densely spaced sampling times at the beginning of infection and sparse sampling times later, leads to decreasing successive escape rate estimates, even when there were no selection differences among alleles. These predictions are supported by empirical data from one HIV-infected patient. Thus, interference could explain why later escapes are slower. Considering escape mutations in isolation, neglecting their genetic linkage, conceals the underlying haplotype dynamics and can affect the estimation of the selective pressure exerted by CD8^+^ cells. In systems in which multiple escape mutations appear, the occurrence of interference dynamics should be assessed by measuring the linkage between different escape mutations.

## 1 Introduction

CD8^+^ T cell responses exert strong selection pressures on Human Immunodeficiency Virus (HIV). Evidence for the importance of CD8^+^ T cells stems from a variety of observations: first, there are associations between set-point viral load and host HLA alleles (1); second, CD8^+^ cell depletion in non-human primates leads to a sharp increase of virus loads (2); lastly, viral mutants that escape CD8^+^ T cell control can have a large fitness cost (3–9). The selective pressure exerted by CD8^+^ T cell responses has been quantified from the growth rate advantage of these escape mutants (10–12).

More recently, studies based on the analysis of the entire viral genome revealed escape in multiple epitopes targeted by CD8^+^ T cell responses (13, 14). In particular, escape mutations have been found to sequentially fixate in up to eight epitopes. A mathematical analysis of these data shows that late-emerging escape mutants outgrow the resident virus population more slowly than early escapes (11, 14–18). We refer to this pattern as escape rate decrease (ERD).

Escape rate decrease has been assumed to have a biological basis, arising from either variation in fitness costs (15) or a decrease of fitness advantages of escape mutants during the course of infection (15, 17–19). But it could also be due to complex dynamical interactions between escape mutations in different epitopes. The complexity arises from the fact that two similarly beneficial mutations rarely arise simultaneously on one genome. Rather, they arise on different genomes which, after outcompeting the wild type strain, enter a state of competition. This state is eventually resolved by one mutation going to fixation by chance, or by one of the strains acquiring the other beneficial mutation. In this scenario, the fixation of one of the advantageous mutations takes longer than its fitness advantage predicts. In population genetics, this complex dynamics is called *interference* (20–23).

**Figure 1:**
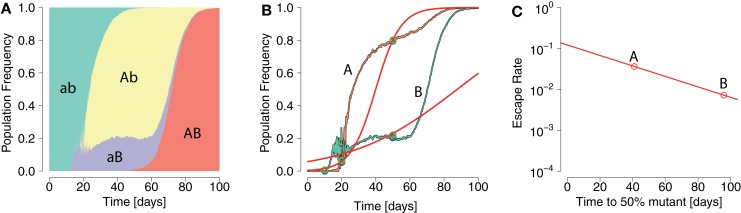
**Pattern of ERD emerging from repeated application of logistic model fits in an interference scenario**. **(A)** Population frequencies of two-locus system haplotypes display interference. The wildtype ab (green area) gives rise to two beneficial single mutants Ab and aB (yellow and violet areas, respectively). **(B)** Fixation patterns of the beneficial alleles A (orange line) and B (green line). Samples of frequencies of A and B are taken at times 10, 20, and 50 days (blue points). Logistic model fits (red lines) are laid through sample points with an added noise (green points). **(C)** From each logistic model fit the escape time and escape rate are calculated. A pattern of ERD is generated due to interference.

Figure 1 shows how interference can affect the interpretation of ERD in early HIV infection. Describing the escape dynamics with models that reduce the dynamics to the competition between two types only – a wildtype and a single escape mutation – and neglect interference, misrepresents the selection pressures at work, and may lead to biased estimates of their strength.

In this study, we investigated under which circumstances genetic interference arises and may thus lead to misinterpretation of pattern of escape rate changes in systems with two loci. We adopted a modeling approach, building on well-established research on the dynamics of HIV within its host (11, 17, 24–27). To this end, we developed a virus dynamics model, in which viruses possess multiple epitopes and can escape from CD8^+^ T cells directed against them. Fitness costs associated with escape mutations are neglected. The model builds on well-established work (11, 17, 24–27), and is stochastic to describe mutation, extinction, and fixation of virus strains adequately, and allows recombination of viruses.

We found that interference emerges mainly when CD8^+^ T cell responses coincide and are similarly strong, but only in systems with a high level of stochasticity. This interference leads to ERD if the virus population was sampled more often early than late, a scheme commonly adopted in empirical studies (14). We tested these predictions in early-infection data from an HIV-positive patient obtained by Henn et al. (16) and subsequently reconstructed to haplotypes (28).

## 2 Materials and Methods

Here, we extend the model of Althaus and De Boer (17), which is in turn based on earlier work (11, 24–27). In our model, a viral strain **i** is assumed to present *n* different viral epitopes to the hosts’ immune system. The strain is represented by a string of binary digits, where 1 at the *j*^th^ entry signifies the presence of an escape mutation in the *j*^th^ epitope. A 0 at the same entry signifies no mutation, and the strains is recognized by epitope-specific immune responses *E_j_*(*t*). The model equations are:

(1) $$\begin{array}{ll} \frac{d}{\mathrm{dt}}T & =\sigma -d_{T}T-\sum_{\text{i}}\frac{\beta \mathrm{Tp}P_{i}}{h_{\beta }+T} \end{array}$$

(2) $$\begin{array}{llll} \frac{d}{\mathrm{dt}}I_{\text{i}} & =\frac{\beta \mathrm{Tp}P_{i}}{h_{\beta }+T}-dI_{\text{i}}-\gamma I_{\text{i}}+\sum_{\text{x}}\left(m_{\text{xi}}I_{\text{x}}-m_{\text{ix}}I_{i}\right)\; & & \;\\ & \;+r\cdot t_{R}\cdot I_{\mathrm{tot}}\left(\sum_{x,y\in Q\left(i\right)}\frac{P_{x}P_{y}}{P_{\mathrm{tot}}^{2}}\frac{1}{2^{d\left(x,y\right)}}\omega^{\prime }\left(x,y,i\right)\right.\left.-\sum_{x}\frac{2P_{x}P_{i}}{P_{\mathrm{tot}}^{2}}\frac{2^{d\left(x,i\right)}-1}{2^{d\left(x,i\right)}}\left(-\omega^{\prime }\left(x,i,i\right)\right)\right) \end{array}$$

(3) $$\frac{d}{\mathrm{dt}}P_{\text{i}}=\gamma I_{\text{i}}+\delta P_{\text{i}}-k\sum_{j}^{n}\left(\frac{a_{ji}E_{j}}{h_{k}+\sum_{\text{x}}a_{jx}P_{\text{x}}+\sum_{s}^{n}a_{s\text{i}}E_{s}}\right)P_{\text{i}}.$$

### 2.1 Target cells

We assume a compartment of CD4^+^ target cells *T*, which is replenished at rate *σ* and gets naturally depleted at a rate *d_T_* per cell. Virions of type **i**, *V*_**i**_, will infect target cells at a rate $\frac{\beta TV_{i}}{h\beta \;+\;T}$ and produce infected cells (*I_i_*), where β is the maximum infection rate per day for a virus particle, and *h*_β_ is the target cell density where the infection rate is half-maximal (17, 26). Viral load and productively infected cells of type **i** (*P*_**i**_) are connected by $\frac{dV_{i}}{\mathrm{dt}}\;=\;fP_{i}-d_{V}V_{i}$. We assume that *V*_**i**_ and *P*_**i**_ are fast coupling. Hence, *V*_**i**_ = *pP*_**i**_, where $p=\frac{f}{d_{V}}$ is the net production rate per cell.

### 2.2 Infected cells

Target cells infected with strain **i** (*I*_**i**_) die at rate *d* and enter an eclipse phase at rate *γ*, after which they become productively infected.

#### 2.2.1 Mutation

Cells infected with strains **x** are converted to cells infected with strain **y** at rate *m*_**xy**_. *m*_**xy**_ is the locus-wise product of the probabilities for an epitope in strain **x** to be mutated into the corresponding epitope in strain **y**. We distinguish between forward mutations and reverse mutations. Forward mutations change a 0 allele into a 1. Reverse mutations do the opposite. An epitope is assumed to consist of about *m* = 8 codons. The mutation rate is 3 ⋅ 10^−5^ per bp per replication (29). For an escape epitope to emerge, this amounts to a rate of ≈1 × 10^−4^ per epitope per replication. The reverse mutation rate was set to ≈5 × 10^−7^ per epitope per replication (see Supplementary Material).

#### 2.2.2 Recombination

Cells infected with strain **i** can arise and be lost by recombination. Both processes are assumed to occur at the same baseline recombination rate *r* and to be proportional to the fraction of co-infected cells in the population *t_R_* ≈ 5 ⋅ 10^−3^, (30–34). $I_{\mathrm{tot}}=\sum_{i}I_{i}$ is the total number of infected cells. We chose the baseline recombination rate *r* to incorporate those rates which do not directly depend on the strain frequencies or types.

The terms within brackets in the recombination term in Eq. 2 deal with probabilities that depend on the strain frequencies. The first sum encompasses recombination events that increase *I*_**i**_. All pairs (**x**, **y**) that can recombine into **i**, *Q*(**i**), are considered. Each such pair (**x**, **y**) co-infected a cell with probability $\frac{P_{x}P_{y}}{P_{\mathrm{tot}}^{2}},$ where $P_{\mathrm{tot}}=\sum_{j}P_{j}$. Out of a pair (**x**, **y**), 2*^d^*^(^**^x^**^,^**^y^**^)^ distinct recombinant offspring types can potentially arise, where *d*(**x**, **y**) denotes the Hamming distance between two strains. Hence, the probability of **i** to be the recombinant offspring is 1/2*^d^*^(^**^x^**^,^**^y^**^)^, where *d*(**x**, **y**) denotes the Hamming distance between two strains.

Infected cell numbers should remain unaltered by the action recombination, since recombination only reshuffles alleles. We account for this by using the weights *ω*′(**x**, **y**, **i**). For example, if the parent pair (**x**, **y**) is lost by recombination to **i**, the net number of cells infected with **i** needs to be multiplied by *ω*′(**x**, **y**, **i**) = 2. However, if one of the parent strains (**x**, **y**) is identical to **i**, the net increase will only be of one. Thus, we defined *ω*′(**x**, **y**, **i**) = 2 − **1_x=i_** − **1_y=i_**, where **1_y=i_** is one if **y** = **i** and zero otherwise (see Supplementary Material for details on *ω*′).

The second type of events that can decrease *I*_**i**_ are those in which the strain **i** can recombine with any other into a strain different from **i**. Pairs can be formed with all other strains, including itself with probability $\frac{2P_{x}P_{i}}{P_{\mathrm{tot}}^{2}}.$ Such an event needs to be weighted with the probability that **i** will yield offspring distinct to itself: $\frac{2^{d(\text{x},\text{i})}-1}{2^{d(\text{x},\text{i})}}$. Again, in order to keep infected cell numbers unaffected by recombination, the weight −*ω*′(**x**, **i**, **i**) is factored in. Note that the factor −*ω*′(**x**, **i**, **i**), becomes positive in these circumstances when using the above definition of *ω*′ [see Supplementary Material, proportionality to linkage disequilibrium (LD) for details].

The baseline recombination rate *r* incorporates several rates and probabilities. These include the probability for co-packaging two parent strains correctly, the number of newly infected cells produced by a single infected cell, the template switching rate and an assumption about the average distance between escape mutations on the genome. We set *r* ≈ 1.4 ⋅ 10^−4^ day^−1^ for our simulations (see Figure S4 in Supplementary Material).

### 2.3 Productively infected cells

Productively infected cells infected with a strain **i** are generated at rate *γ* from the eclipsed population *I*_**i**_, and die with rate δ. The CD8^+^ T cells specific to the epitope *j*, *E_j_*, clear productively infected cells whose epitopes they recognize maximally at rate *k*, with *h_k_* the Michaelis–Menten constants (17). The coefficients *a*_*j*__**i**_ are 1 if **i** has a zero at its *j*^th^ position, and zero otherwise. They thereby encode the recognition of non-escape epitopes by the CD8^+^ T cells.

### 2.4 CD8^+^ T cells

Unlike previous models describing CD8^+^ T cell escape (17, 26), our model does not dynamically link the immune response to the level of viral antigen. In our model, *E_j_*(*t*) is a numerically constructed fixed time course of the CD8^+^ T cells of type *j*, consistent with a program-type dynamics established for CD8^+^ T cell responses against infections in mice (35–40).

In order to attain a descriptive CD8^+^ T cell function we combined two exponential functions and a constant value. The default CD8^+^ T cell-function starts growing from a single cell at time *t* = 0 at a rate of *r_c_* = 0.9 per day (26, 41), until it reaches an upper limit *C*, which is 1.5 logs larger than the final level value $K:C=10^{\log_{10}\left(K\right)+1.5}$ (35). Once *C* has been reached, the function declines exponentially at a rate of 0.4 *r_c_*, until it reaches *K*. For larger time values, it remains at *K*. In this study, we restricted the *final levels K* to values of the order 10^7^, in accordance with (26, 35, 36).

### 2.5 Implementation

For all simulations described in this paper, we used parameters as shown in Table 1. To implement the dynamics we used the R language for statistical computing (42) and *adaptivetau* (43) for the simulation of Eq. 1 by Gillespie algorithm. The rates of the Gillespie algorithm are the terms on the right-hand side of the ordinary differential equations Eq. 1.

**Table 1:** **Parameter values employed for simulations**.

Parameter	Description	Value
σ	Replenishment rate of target cells *T* (44)	10^8^ cells/day
*d_T_*	Natural rate of target cell death (45, 46)	10^−2^ day^−1^
*p*	Net virion production rate per productively infected cell (47, 48)	10^4^ virions/day
β	Maximum infection rate per day for a virus particle	5.5 ⋅ 10^−4^ 1/day
*h*_β_	Target cell number at which infection rate is half-maximal (17)	5 ⋅ 10^7^ cells
*d*	Natural rate of infected cell death (26, 49)	2 ⋅ 10^−2^ 1/day
γ	Transition rate to productively infected cells (eclipse phase) (50–53)	1 1/day
δ	Natural rate of productively infected cell death (25, 54, 55)	1 1/day
*k*	Maximum killing efficiency (56)^a^	50 1/day
*h_k_*	Cell number at which killing rate is half-maximal (17)	10^9^ cells
μ	Viral mutation rate (29)	3 ⋅ 10^−5^ 1/bp replication
*r*	Recombination rate (32, 57–59)	1.4 ⋅ 10^−4^ 1/day

*^a^We deliberately omitted escape-based estimates here (10–12, 14)*.

### 2.6 Stochasticity induced by system rescaling

Human immunodeficiency virus large census population size does not imply a small role of stochastic effects in its dynamics (60). Hence, we implemented methods to rescale the model system to match a population size of 10^6^ (see Supplementary Material). By downsizing or magnifying a system we refer to transforming the system under consideration *S*, with its variables *T*, *I_i_*, *P_i_* into a system downsized by a factor *a*, *S_a_*, with corresponding variables *T_a_*, *I_i,a_*, *P_i,a_*. Under a deterministic framework, the ratios between time courses of the downsized variables and the corresponding original system variables are 1/*a*. To rescale the responses *E_j_* with *a*, we left the growth and contraction rates unchanged, but modified *K* such that *K_a_* = *a* ⋅ *K*.

## 3 Results

### 3.1 Virus dynamics model reproduces escape dynamics

**Figure 2:**
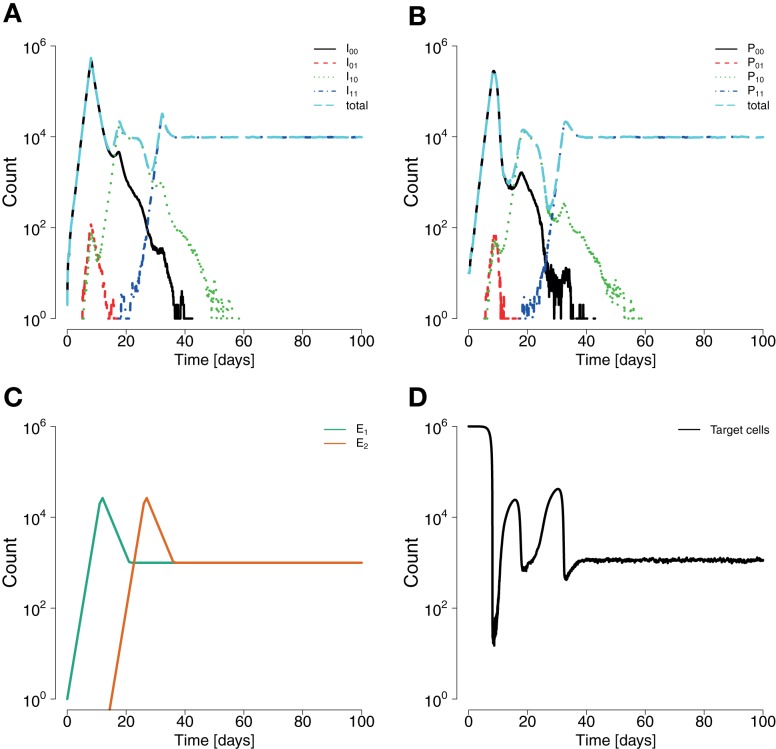
**Example for a simulation run showing sequential escapes in a scaled down two-locus two-allele system**. **(A)** Time course of the number of non-productively infected cells by strain types show sequential transitions from the wildtype to a single escape mutant to a double mutant. **(B)** Analogous situation for the time courses of productively infected cells. Productively infected cells are cleared by epitope-specific CD8^+^ T cell action. The mounted immune response leads to a transitory decrease of the total number of productively infected cells. **(C)** The CD8^+^ functions *E*_1_ and *E*_2_ start at 0 and 15 days, respectively, with a final value of 10^7^. The mounting of the immune response coincides with the temporary reduction of infected and productively infected cell numbers. **(D)** The killing of productively infected cells causes the transitory reduction of produced virions, temporarily reducing net new infections, and releasing target cells. Parameters are as given in Table 1.

The model presented here reproduces basic empirically observed aspects of HIV/SIV dynamics (Figure 2). In our model, the initial growth of the virus has been gaged to 1.2 ± 0.1 per day in accordance with (61). Consistent with clinical and experimental data (46, 62, 63), the viral load peaks around day 20 after infection. In line with (64), there are estimated to be ≈10^8^ HIV-infected target cells at the viral set point. This value is about one to two orders of magnitude below peak viremia, in accordance with Ref. (65–67). The virus can only go extinct due to stochastic effects in the beginning of infection. Lastly, the model can also realistically reproduce the simultaneous emergence of distinct viral escape mutations, as well as the generation of double escape mutants by mutation or recombination.

### 3.2 Cumulative linkage disequilibrium as a measure for interference

**Figure 3:**
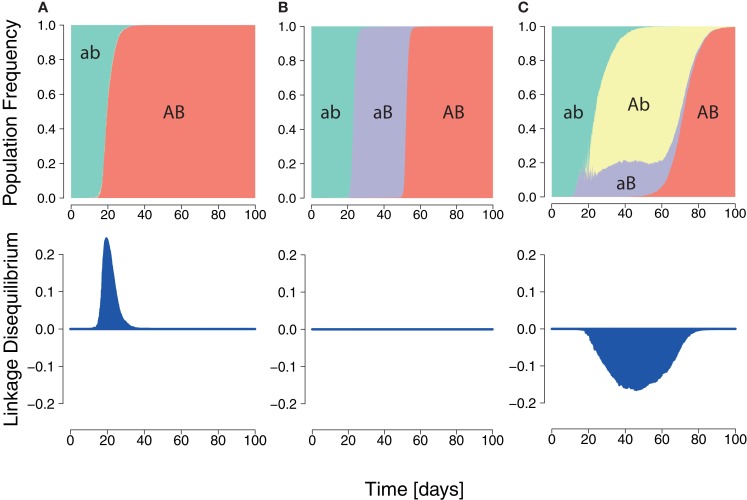
**Three HIV dynamics scenarios and the cumulative LD**. **(A)** The wildtype population is replaced by a double mutant. A positive LD is generated during the replacement leading to positive cumulative LD. **(B)** Succession of wildtype, single mutant, and double mutant. LD remains zero for each transition. **(C)** Wildtype is replaced by two single mutants, which are in turn outcompeted by the double mutant. The longer the single mutants coexist, the more negative the cumulative LD value will be.

In order to quantify the *expressed interference* between viral escape strains during infection, we used the population genetics measure of LD. In our two-epitope system (Figure 3), the LD is *D* = *p_ab_p_AB_* − *p_Ab_p_aB_*, where *p_ab_* is the frequency of the wildtype, *p_Ab_* and *p_aB_* are the frequencies of single mutants, and *p_AB_* is the frequency of the double escape strain (where *A* and *B* are strongly advantageous).

In general, the wildtype is first replaced by single mutants, which are then outcompeted by the double mutant (Figure 3) (22, 68, 69). This dynamics is characterized by the duration of the intermediate phase of single mutant dominance and by the diversity in single mutants.

A quantitative measure of expressed interference of the dynamics has to behave appropriately when duration and diversity change. Firstly, the longer two single mutants coexist, the higher the value of expressed interference should be (see Figure S1 in Supplementary Material). Second, the higher the diversity during the state of stasis, the higher the interference measure should be (see Figure S2 in Supplementary Material). The cumulative LD satisfies both of these requirements. The term *cumulative* refers to the integral of the LD over time. This measure is also well-behaved in standard population genetics models (see Figure S3 in Supplementary Material).

Negative values of the cumulative LD specifically characterize interference in a regime where selection is much stronger than recombination, and not other types of dynamics. Dynamics in which escape mutations sequentially fixate, are characterized by zero LD.

### 3.3 Similarly strong and synchronously elicited CD8^+^ T cell functions facilitate the appearance of interference

In our model, we considered two CD8^+^ T cell responses, each recognizing one of two epitopes. We investigated how differences in the strength and relative timing (see Materials and Methods) of CD8^+^ T cell functions affect the HIV dynamics. Each CD8^+^ T cell time course could assume two different values for its strength: *K_j_* ∈ {1 ⋅ 10^7^, 7 ⋅ 10^6^}, where j ∈ {1, 2} denotes the order of elicitation. The first response was set to start at *t* = 0, and the second started with a delay of 0, 5, 10, 15, 20, 25, or 30 days. Each combination of CD8^+^ T cell function pairs was simulated 100 times.

We consider two levels of stochasticity here: a low and a high level (see Materials and Methods). The low level is displayed in our simulations at census population size of about 10^8^ at set point with parameters as in Table 1. The high level of stochasticity arises when we scale the population sizes by a factor *a* = 10^−4^ (see Supplementary Material), consistent with empirical estimates of HIV’s effective population size (70, 71).

#### 3.3.1 Negative cumulative LD most pronounced at near-identical CD8^+^ T cell responses

**Figure 4:**
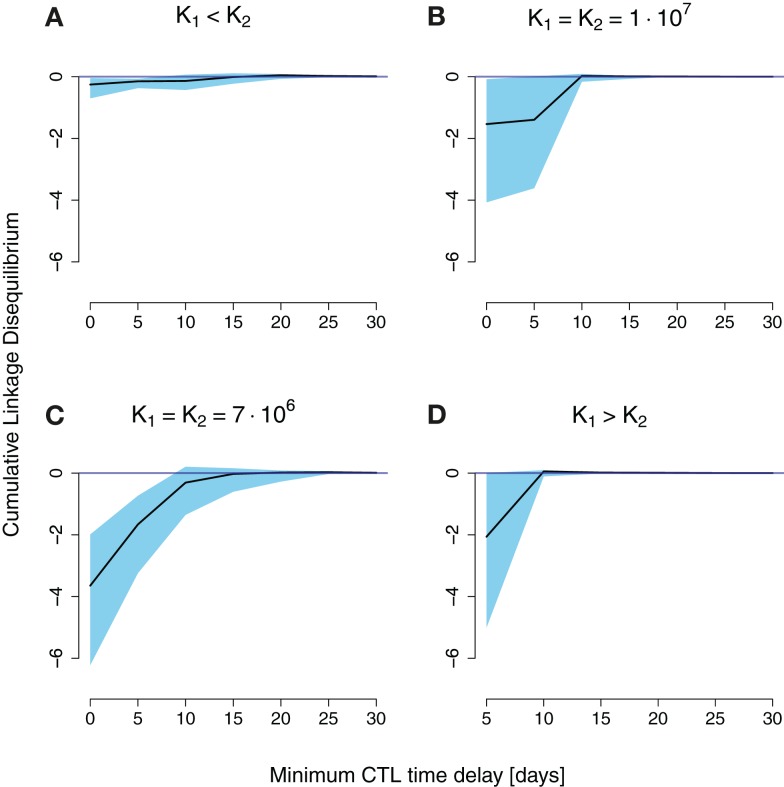
**Cumulative LD values for simulation runs of a downsized two-locus system (*a* = 10^−4^) differing in CD8^+^ T cell function strength and timing**. The *x*-axis denotes the time delay the second immune response has to the first. The black line is the median of 100 simulations, the upper and lower end of the blue-shaded area are the 75 and 25 percentiles of all measured simulation runs, respectively. *K_1_* and *K_2_* denote the final values for the first and the second immune responses, respectively. **(A)** Little cumulative LD is generated for *K_1_* < *K_2_*. Pronounced negative cumulative LD values are attained for nearly equally spaced CD8^+^ T cell curves in **(B,C)**. **(D)** Shows an increase in interference at a delay of 5 days.

To investigate under which circumstances interference between escape mutation arises we calculated the cumulative LD for different combinations of strength and timing of CD8^+^ T cell responses, and levels of stochasticity. For the unscaled system with low stochasticity, simulations showed positive cumulative LD, indicating the immediate emergence of double escapes (see Figure S5 in Supplementary Material). In contrast, Figures 4A–D show negative values of cumulative LD for the downsized system with high stochasticity, indicating interference. Interference is particularly likely to occur when CD8^+^ T cell responses are nearly synchronous and nearly equally strong.

The emergence of interference at high stochasticity levels can be explained in terms of population genetics. The level of stochasticity or drift is inversely related to the population size *N*. The expected number of double mutants is $N\mu_{b}^{2}$ per generation, where μ*_b_* ≈ 10^−4^ is the rate of beneficial mutations per epitope and generation. If this number is larger than the establishment size 1/2S ≈ 1 at the onset of selection, the double mutant is assumed to go to fixation (*S* is the selective advantage of one beneficial mutation). This is the case at low levels of stochasticity (*N* ≈ 10^9^), which leads to positive cumulative LD. Conversely, high levels of stochasticity will delay the emergence of double mutants, entailing interference between single mutants. Note that this behavior is dissimilar to the standard behavior without a preceding neutral phase, where larger population sizes cause more interference.

The reasons for the general pattern of negative cumulative LD are intuitively clear. First, synchronous (time delay zero), but unequally powerful CD8^+^ T cell functions favor one of the single escape strains, leading to its fast fixation (Figures 4A,D). This dynamics of sequential escape produces no substantial cumulative LD, and no interference.

Second, CD8^+^ T cell responses that are elicited far apart in time induce practically no interaction between haplotypes (Figures 4B,C). The earlier immune response will select for a first escape mutant, and the second elicited immune response will select for the double escape mutant. Again, the escape dynamics is sequential, which leaves no trace in the cumulative LD.

Third, higher selective pressures (higher *K*’s) will decrease the time of emergence as well as the fixation time of double mutants. The coexistence time of single mutants will thereby be reduced. Therefore, the cumulative LD, which scales roughly as the coexistence time, will be smaller compared to lower selective pressures (Figures 4B,C).

There is one exception to the general pattern: when the first immune response is stronger than the second *K*_1_ > *K*_2_, and precedes it by 5 days, substantial amounts of negative cumulative LD are generated. This result arises through the complex interplay between the timing and strength of the two responses in this scenario. With a delay of 5 days, the total population of productively infected cells is at a local minimum at about the time when the action of the second response is at its peak. Due to the contracting dynamics of the CD8^+^ T cell responses, the second response is stronger than the first at that time point, even for *K*_1_ > *K*_2_. The difference between the responses at that time point is small enough to allow for the emergence of interference.

#### 3.3.2 Escape rate decrease

For each simulation, we calculated the *escape time* τ_50_ and *escape rate* ε from the frequency of both escape mutations (disregarding their linkage), mimicking the sampling schedule and data collection of previous studies (11, 14–16) (see Supplementary Material, Sampling Methods and Fitting section).

The estimated escape rates were in good accordance with common values for escape rates during early-infection. For example, escape rates at high interference conditions (Figure 4C) were about 0.04 day^−1^ in the median, 0.02 day^−1^ for the 2.5%-quantile, and 0.17 day^−1^ for the 97.5%-quantile.

With the values *τ*_50_ and ε for each escape mutation, we calculated the successive ERD in each simulation. This was done by fitting a linear regression log_10_(ε) = *a* + *b* ⋅ *τ*_50_, as in Ref. (15). The slope of the regression *b* is termed ERD value. Negative ERD values indicate that later escapes are slower.

For small time delays, the ERD values are between −0.01 and 0 (see Figure S6 in Supplementary Material). In Ref. (14, 15), ERD values inferred from escapes with *τ*_50_ within the first 2 years, are about −0.006 in patient CH44, −0.008 in patient CH77 and −0.01 in patient CH58 [based on data in Supplementary Material of Ref. (15)].

### 3.4 Cumulative LD is associated with escape rate decrease

To investigate the effects of interference on the ERD values we focused on those time delays shown in Figure 4 that showed substantial interference.

**Figure 5:**
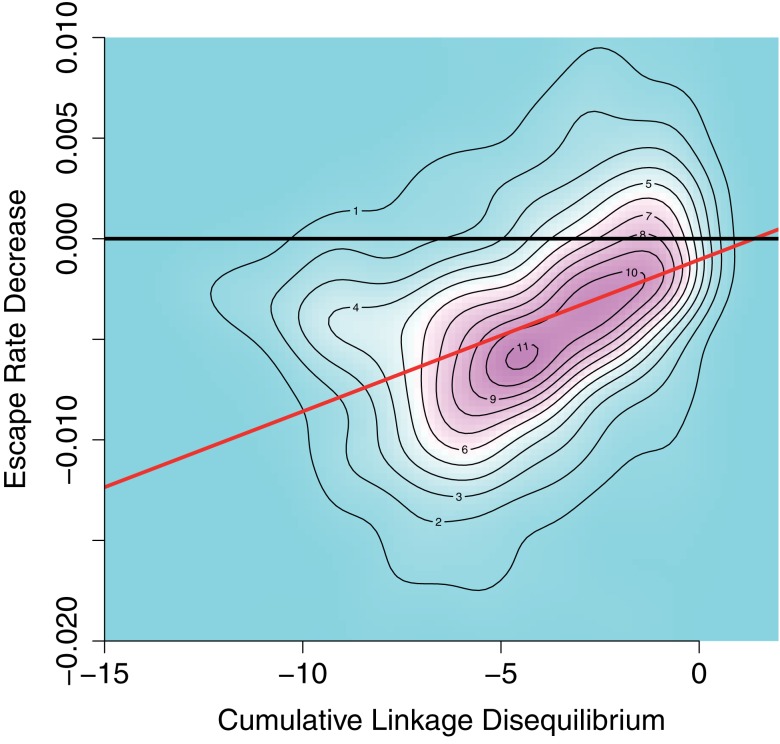
**Density plot of negative cumulative LD versus ERD values for 1000 simulations runs for equal CD8^+^ T cell final values of *K*_1_ = *K*_2_ = 7 ⋅ 10^6^ and zero time delay between the elicitation of the CD8^+^ T cell functions**. Positive cumulative LD values were ignored. Black line: base line through the origin. Red line: Theil–Sen estimator fit on data. The density distribution is compressed along a line of positive slope, indicating a positive association between interference and ERD values.

Figure 5 shows the association between cumulative LD and ERD values in 1000 simulations run for CD8^+^ T cell functions with equal final levels *K*_1_ = *K*_2_ = 7 ⋅ 10^6^ and no time delay. In the plot, simulations which lead to a cumulative LD larger than zero (about 11% of simulations) have been removed in order to assess the effects of interference only. The density distribution shows a clustering of simulation results along a line of positive slope. The distribution is compressed along that line.

A Theil–Sen estimator fit to the data (red line) yields a slope of 8 × 10^−4^ with confidence intervals (6 × 10^−4^, 9 × 10^−4^) for the 2.5 and 97.5 percentiles, respectively. This indicates an association of interference and ERD. The pattern shown in Figure 5 can also be identified in the other case with identical CD8^+^ T cell final levels (*K*_1_ = *K*_2_ = 10^7^) with no time delay (see Figure S7A in Supplementary Material).

We considered other combinations of final levels and time delays with median cumulative LD below minus one in the down-scaled system. As time delays increase, in simulations with CD8^+^ T cell responses of equal strength we observe the appearance of a density peak alongside the interference, centered at about zero LD and negative ERD values. This peak indicates the appearance of a different mode of escapes. These escapes are sequential and show no interference. As expected, this second mode eventually replaces the interference pattern as the time delay increases (see Figure S7 in Supplementary Material).

In the escapes of this second mode, the negative ERD values in the absence of interference emerge due to an artifact of the sampling scheme employed. However, the associations found between cumulative LD and ERD values in Figure 5 indicate that a causal relation must be present, because the sampling scheme is identical in each simulation. Whatever offset to the ERD value might be contributed by the sampling scheme, it cannot explain these associations.

### 3.5 Possible instance of escape rate decrease due to interference

To investigate one possible instance of interference and its effects on escape rates, we analyzed haplotype data obtained by deep sequencing from a single patient (subject 9213) infected with HIV (16). In this study, blood samples were taken at days 0, 3, 59, 165, 476, and 1543 after infection was determined. Haplotypes were subsequently reconstructed from these sequence data by Pandit et al. (28).

These data are of particular interest to test the effects predicted by our model because CTL responses specific to two epitopes, *Nef A24-RW8* and *Vif B38-WI9* were similarly strong. At day 59, these responses differed only about 10% in terms of Elispot responses in Spot Forming Cells (SFC) per million Peripheral Blood Mononuclear Cells (PBMC) [see Supplementary Information in Ref. (16)].

**Figure 6:**
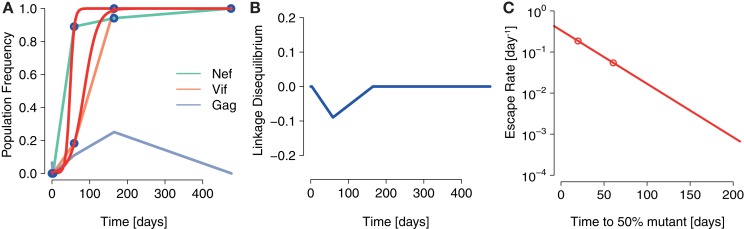
**A possible instance of interference generating ERD in Ref. (**16). **(A)** The fits of the logistic escape model as used in Ref. (15) to the sample points of escape mutant frequency. Escape mutations appear in the epitopes *Nef A24-RW8* and *Vif B38-WI9* as a consequence of two, nearly equally potent CTL responses specific to these epitopes. **(B)** The LD between *Nef A24-RW8* and *Vif B38-WI9* becomes negative at one of the time points sampled (connected by blue line). To calculate LD, we constructed a contingency table with the frequencies of wildtype (no mutations in any epitope), single mutants (escape mutation in one epitope) and the double mutant (escape mutations in both *Nef* and *Vif*) reconstructed in Ref. (28) and measured *D* as defined above. **(C)** The inferred escape rates of the escape mutations in *Nef* and *Vif* show ERD.

To test the predictions of our model, we fitted logistic escape functions to the data and measured LD and escape rates. Figure 6 shows the fitted escape curves on the mutant frequencies of *Nef* and *Vif*, respectively, in Figure 6A. At day 59 the inferred LD in these data are *D* = −0.09, as shown in Figure 6B. As shown in Figure 6C, ERD is clear in the escape of these two mutations.

The results of this analysis are in accordance with the notion that interference was acting to delay the emergence of escapes and to reduce their escape rates.

## 4 Discussion

In this paper, the main focus was the interference of viral strains and its role in the escape dynamics. We identified signals of interference under very specific conditions: in systems with high levels of stochasticity and highly synchronized and comparably strong CD8^+^ T cell responses, the generation of interference is facilitated. These conditions are often satisfied in empirically observed HIV dynamics (35, 37, 70–72). Furthermore, when sampling at times typical for empirical studies, increasing interference decreases ERD values. Lastly, we also tested these predictions in one instance of two equally strong CTL responses elicited against HIV in a patient (16). In the data, a signal for interference was accompanied by ERD.

We restricted the simulations to two-locus systems, while ERD was inferred from empirical data involving more escape variants. Our results are consistent with theoretical findings for two-locus systems in the context of population genetics (73). Intuitively, the principle that under interference the fast escape of one allele implies the slow escape of competing alleles with similar fitness should also hold in systems with more than two loci. Interference effects for systems with more loci are expected to be stronger (17, 19) and are likely to have a stabilizing impact on ERD (17) (Figure 5). We thus hypothesize that the pattern of ERD is preserved in HIV dynamics models with more than two epitopes showing interference. How interference between multiple beneficial mutations at distinct epitopes is connected to multi-locus linkage disequilibria is an open question. To our knowledge, there exists no consensus on how to define LD for more than three loci (74–76). This would be an interesting subject for further investigation.

Furthermore, we ignored potential fitness costs of escape mutations in our simulations. They can be safely neglected if they are compensated at faster rates than the fixation times of beneficial mutants. Only if fitness costs differ substantially between escape mutants do we expect that their explicit consideration will alter the role of interference.

These results have to be interpreted in the larger context of estimating the selective pressures that immune responses exert on the virus population. These selection pressures are often inferred from the growth advantage of mutants that escaped the immune response – the rate of escape. Estimating these rates of escape using models that neglect the complex genetical interactions between escape strains has revealed the pattern of ERD central to our study. Our study shows that caution is warranted when drawing conclusions from this pattern about the selection pressures at work.

Very recently, Pandit et al. identified a clear instance of clonal interference in HIV between mutations within the same epitope as well as between epitopes (28). The relevance of interference during early HIV is further supported by “epitope shattering,” where a founder strain can diversify into an array of strains with distinct escape mutations at the same epitope (77). Leviyang studied such mutational pathways in data presented in Ref. (78), focusing on competition between intra-epitope escape mutations (79). O’Connor also reported the coexistence of escape mutations within the same epitope in SIV-infected Mauritian cynomolgus macaques (80).

In contrast to our investigations, other studies expect interference effects to be negligible. Da Silva modeled early HIV infection with a Wright–Fisher process incorporating weakening CD8^+^ T cell responses (81). He concluded that due to the transmission bottleneck the effective population size of HIV should remain at low levels (*N_e_* ≈ 10^2^) throughout early-infection, thus preventing interference.

Kessinger et al. (82) estimated escape rates of the HIV data presented in Goonetilleke et al. (14) by employing multi-epitope models of HIV. To do this, they imposed a scheme of sequential escapes on their model. With that scheme, the escape rate estimates were substantially higher than in other studies.

Very recently, Ganusov et al. presented stochastic simulations of a multi-epitope model of HIV infection with recombination (19). In that paper, the bias interference effects introduce in escape rate estimates is also discussed. Escape rate estimates are heavily underestimated at low sample sizes (≈20 samples), but improve at sample sizes of about 200. They also find that in stochastic simulations, escapes are delayed compared to deterministic escapes, especially for low recombination rates. These theoretical results strongly support the notion that current estimation methods might be inappropriate tools for escape rate inference under interference regimes.

Our study makes a few testable predictions. Whether interference is involved in the generation of ERD can be assessed by measuring the LD between HIV haplotypes over time. This requires sequencing that either retains linkage information, or the reconstruction of haplotypes using bioinformatic methods (83, 84). Sustained negative linkage disequilibria would be indicative of interference. In this case, interference needs to be corrected before escape rates can be related to selection pressures. After this correction, the estimates of the selection pressures should be higher than previously estimated by means of logistic curve fitting. Furthermore, interference should be enhanced in mutations of epitopes in close proximity in the genome, especially mutations within the same epitopes.

## Conflict of Interest Statement

The authors declare that the research was conducted in the absence of any commercial or financial relationships that could be construed as a potential conflict of interest.

## Supplementary Material

The Supplementary Material for this article can be found online at http://www.frontiersin.org/Journal/10.3389/fimmu.2014.00661/abstract

## References

[1] Goulder P Watkins D HIV and SIV CTL escape: implications for vaccine design. Nat Rev Immunol (2004) 4(8):630–40 10.1038/nri1417 15286729

[2] Schmitz J Kuroda M Santra S Sasseville V Simon M Lifton M Control of viremia in simian immunodeficiency virus infection by CD8+ lymphocytes. Science (1999) 283(5403):857. 10.1126/science.283.5403.857 9933172

[3] Friedrich TC Dodds EJ Yant LJ Vojnov L Rudersdorf R Cullen C Reversion of CTL escape-variant immunodeficiency viruses in vivo. Nat Med (2004) 10(3):275–81. 10.1038/nm998 14966520

[4] Barouch DH Powers J Truitt DM Kishko MG Arthur JC Peyerl FW Dynamic immune responses maintain cytotoxic T lymphocyte epitope mutations in transmitted simian immunodeficiency virus variants. Nat Immunol (2005) 6(3):247–52. 10.1038/ni1167 15685174

[5] Kent SJ Fernandez CS Jane Dale C Davenport MP. Reversion of immune escape HIV variants upon transmission: insights into effective viral immunity. Trends Microbiol (2005) 13(6):243–6. 10.1016/j.tim.2005.03.011 15936652

[6] Peut V Kent SJ. Fitness constraints on immune escape from HIV: implications of envelope as a target for both HIV-specific T cells and antibody. Curr HIV Res (2006) 4(2):191. 10.2174/157016206776055110 16611057

[7] Crawford H Prado JG Leslie A Hué S Honeyborne I Reddy S Compensatory mutation partially restores fitness and delays reversion of escape mutation within the immunodominant HLA-B* 5703-restricted Gag epitope in chronic human immunodeficiency virus type 1 infection. J Virol (2007) 81(15):8346–51 10.1128/JVI.00465-07 17507468 PMC1951305

[8] Frater AJ Brown H Oxenius A Günthard H Hirschel B Robinson N Effective T-cell responses select human immunodeficiency virus mutants and slow disease progression. J Virol (2007) 81(12):6742–51. 10.1128/JVI.00022-07 17409157 PMC1900110

[9] Li B Gladden AD Altfeld M Kaldor JM Cooper DA Kelleher AD Rapid reversion of sequence polymorphisms dominates early human immunodeficiency virus type 1 evolution. J Virol (2007) 81(1):193–201. 10.1128/JVI.01231-06 17065207 PMC1797245

[10] Fernandez CS Stratov I De Rose R Walsh K Dale CJ Smith MZ Rapid viral escape at an immunodominant simian-human immunodeficiency virus cytotoxic T-lymphocyte epitope exacts a dramatic fitness cost. J Virol (2005) 79(9):5721–31. 10.1128/JVI.79.9.5721-5731.2005 15827187 PMC1082732

[11] Asquith B Edwards CT Lipsitch M McLean AR. Inefficient cytotoxic T lymphocyte-mediated killing of HIV-1-infected cells in vivo. PLoS Biol (2006) 4(4):e90. 10.1371/journal.pbio.0040090 16515366 PMC1395353

[12] Mandl JN Regoes RR Garber DA Feinberg MB. Estimating the effectiveness of simian immunodeficiency virus-specific CD8+ T cells from the dynamics of viral immune escape. J Virol (2007) 81(21):11982–91. 10.1128/JVI.00946-07 17699572 PMC2168796

[13] Turnbull E Wong M Wang S Wei X Jones N Conrod K Kinetics of expansion of epitope-specific T cell responses during primary HIV-1 infection. J Immunol (2009) 182(11):7131–45. 10.4049/jimmunol.0803658 19454710

[14] Goonetilleke N Liu M Salazar-Gonzalez J Ferrari G Giorgi E Ganusov V The first T cell response to transmitted/founder virus contributes to the control of acute viremia in HIV-1 infection. J Exp Med (2009) 206(6):1253–72. 10.1084/jem.20090365 19487423 PMC2715063

[15] Ganusov VV Goonetilleke N Liu MK Ferrari G Shaw GM McMichael AJ Fitness costs and diversity of the cytotoxic T lymphocyte (CTL) response determine the rate of CTL escape during acute and chronic phases of HIV infection. J Virol (2011) 85(20):10518–28. 10.1128/JVI.00655-11 21835793 PMC3187476

[16] Henn MEA. Whole genome deep sequencing of HIV-1 reveals the impact of early minor variants upon immune recognition during acute infection. PLoS Pathog (2012) 8(3):e1002529. 10.1371/journal.ppat.1002529 22412369 PMC3297584

[17] Althaus CL De Boer RJ. Dynamics of immune escape during HIV/SIV infection. PLoS Comput Biol (2008) 4(7):e1000103. 10.1371/journal.pcbi.1000103 18636096 PMC2423483

[18] van Deutekom HW Wijnker G de Boer RJ. The rate of immune escape vanishes when multiple immune responses control an HIV infection. J Immunol (2013) 191(6):3277–86. 10.4049/jimmunol.1300962 23940274

[19] Ganusov VV Neher RA Perelson AS. Mathematical modeling of escape of HIV from cytotoxic T lymphocyte responses. J Stat Mech (2013) 2013(01):01010. 10.1088/1742-5468/2013/01/P01010 24660019 PMC3961578

[20] Hill W Robertson A The effect of linkage on limits to artificial selection. Genet Res (1966) 8(3):269–94 10.1017/S0016672300010156 5980116

[21] Gerrish P Lenski R. The fate of competing beneficial mutations in an asexual population. Genetica (1998) 102:127–44. 10.1023/A:1017067816551 9720276

[22] Desai MM Fisher DS. Beneficial mutation selection balance and the effect of linkage on positive selection. Genetics (2007) 176(3):1759–98. 10.1534/genetics.106.067678 17483432 PMC1931526

[23] Imhof M Schlötterer C. Fitness effects of advantageous mutations in evolving *Escherichia coli* populations. Proc Natl Acad Sci U S A (2001) 98(3):1113–7. 10.1073/pnas.98.3.1113 11158603 PMC14717

[24] Nowak M May RM Virus Dynamics: Mathematical Principles of Immunology and Virology: Mathematical Principles of Immunology and Virology. New York: Oxford University Press (2000).

[25] Perelson AS Modelling viral and immune system dynamics. Nat Rev Immunol (2002) 2(1):28–36 10.1038/nri700 11905835

[26] De Boer R. Understanding the failure of CD8+ T-cell vaccination against simian/human immunodeficiency virus. J Virol (2007) 81(6):2838–48. 10.1128/JVI.01914-06 17202215 PMC1865966

[27] Fryer HR Frater J Duda A Roberts MG Phillips RE McLean AR Modelling the evolution and spread of HIV immune escape mutants. PLoS Pathog (2010) 6(11):e1001196. 10.1371/journal.ppat.1001196 21124991 PMC2987822

[28] Pandit A de Boer RJ. Reliable reconstruction of HIV-1 whole genome haplotypes reveals clonal interference and genetic hitchhiking among immune escape variants. Retrovirology (2014) 11:56. 10.1186/1742-4690-11-56 24996694 PMC4227095

[29] Mansky LM Temin HM. Lower in vivo mutation rate of human immunodeficiency virus type 1 than that predicted from the fidelity of purified reverse transcriptase. J Virol (1995) 69(8):5087–94. 7541846 10.1128/jvi.69.8.5087-5094.1995 PMC189326

[30] Jung A Maier R Vartanian J-P Bocharov G Jung V Fischer U Recombination: multiply infected spleen cells in HIV patients. Nature (2002) 418(6894):144–144. 10.1038/418144a 12110879

[31] Josefsson L Palmer S Casazza J Ambrozak D Kearney M Shao W Analysis of HIV DNA molecules in paired peripheral blood and lymph node tissue samples from chronically infected patients. Antiviral Therapy. (Vol. 15), London: International Medical Press Ltd (2010). p. A41–41 Available from: http://www.intmedpress.com/serveFile.cfm?sUID=c10a93ca-a12a-4ff9-980d-07561299189b

[32] Neher RA Leitner T. Recombination rate and selection strength in HIV intra-patient evolution. PLoS Comput Biol (2010) 6(1):e1000660. 10.1371/journal.pcbi.1000660 20126527 PMC2813257

[33] Batorsky R Kearney MF Palmer SE Maldarelli F Rouzine IM Coffin JM. Estimate of effective recombination rate and average selection coefficient for HIV in chronic infection. Proc Natl Acad Sci U S A (2011) 108(14):5661–6. 10.1073/pnas.1102036108 21436045 PMC3078368

[34] Mostowy R Kouyos R Fouchet D Bonhoeffer S. The role of recombination for the coevolutionary dynamics of HIV and the immune response. PLoS One (2011) 6(2):e16052. 10.1371/journal.pone.0016052 21364750 PMC3041767

[35] Murali-Krishna K Altman JD Suresh M Sourdive DJ Zajac AJ Miller JD Counting antigen-specific CD8 T cells: a reevaluation of bystander activation during viral infection. Immunity (1998) 8(2):177–87. 10.1016/S1074-7613(00)80470-7 9491999

[36] Ahmed R Gray D. Immunological memory and protective immunity: understanding their relation. Science (1996) 272(5258):54–60. 10.1126/science.272.5258.54 8600537

[37] Antia R Bergstrom CT Pilyugin SS Kaech SM Ahmed R. Models of CD8+ responses: 1. What is the antigen-independent proliferation program. J Theor Biol (2003) 221(4):585–98. 10.1006/jtbi.2003.3208 12713942

[38] Kaech SM Wherry EJ Ahmed R. Effector and memory T-cell differentiation: implications for vaccine development. Nat Rev Immunol (2002) 2(4):251–62. 10.1038/nri778 12001996

[39] Kaech SM Hemby S Kersh E Ahmed R. Molecular and functional profiling of memory CD8 T cell differentiation. Cell (2002) 111(6):837–51. 10.1016/S0092-8674(02)01139-X 12526810

[40] Antia R Ganusov VV Ahmed R. The role of models in understanding CD8+ T-cell memory. Nat Rev Immunol (2005) 5(2):101–11. 10.1038/nri1550 15662368

[41] Davenport MP Ribeiro RM Chao DL Perelson AS. Predicting the impact of a nonsterilizing vaccine against human immunodeficiency virus. J Virol (2004) 78(20):11340–51. 10.1128/JVI.78.20.11340-11351.2004 15452255 PMC521856

[42] Team RDC. R: A Language and Environment for Statistical Computing. Vienna: R Foundation for Statistical Computing (2012).

[43] Johnson P Adaptivetau: Tau-Leaping Stochastic Simulation. R Package Version 0.902 (2011).

[44] Haase AT Henry K Zupancic M Sedgewick G Faust RA Melroe H Quantitative image analysis of HIV-1 infection in lymphoid tissue. Science (1996) 274(5289):985–9. 10.1126/science.274.5289.985 8875941

[45] Mohri H Bonhoeffer S Monard S Perelson AS Ho DD. Rapid turnover of T lymphocytes in SIV-infected rhesus macaques. Science (1998) 279(5354):1223–7. 10.1126/science.279.5354.1223 9469816

[46] Stafford MA Corey L Cao Y Daar ES Ho DD Perelson AS. Modeling plasma virus concentration during primary HIV infection. J Theor Biol (2000) 203(3):285–301. 10.1006/jtbi.2000.1076 10716909

[47] Chen HY Di Mascio M Perelson AS Ho DD Zhang L. Determination of virus burst size in vivo using a single-cycle SIV in rhesus macaques. Proc Natl Acad Sci U S A (2007) 104(48):19079–84. 10.1073/pnas.0707449104 18025463 PMC2141911

[48] De Boer RJ Ribeiro RM Perelson AS. Current estimates for HIV-1 production imply rapid viral clearance in lymphoid tissues. PLoS Comput Biol (2010) 6(9):e1000906. 10.1371/journal.pcbi.1000906 20824126 PMC2932679

[49] Markowitz M Louie M Hurley A Sun E Di Mascio M Perelson AS A novel antiviral intervention results in more accurate assessment of human immunodeficiency virus type 1 replication dynamics and T-cell decay in vivo. J Virol (2003) 77(8):5037–8. 10.1128/JVI.77.8.5037-5038.2003 12663814 PMC152136

[50] Dixit NM Markowitz M Ho DD Perelson AS. Estimates of intracellular delay and average drug efficacy from viral load data of HIV-infected individuals under antiretroviral therapy. Antivir Ther (2004) 9(2):237–46. Available from: http://www.intmedpress.com/journals/avt/abstract.cfm?id=998&pid=88 15134186

[51] Nelson PW Mittler JE Perelson AS. Effect of drug efficacy and the eclipse phase of the viral life cycle on estimates of HIV viral dynamic parameters. J Acquir Immune Defic Syndr (2001) 26(5):405–12. 10.1097/00042560-200104150-00002 11391159

[52] Reddy B Yin J. Quantitative intracellular kinetics of HIV type 1. AIDS Res Hum Retroviruses (1999) 15(3):273–83. 10.1089/088922299311457 10052758

[53] Rouzine IM Sergeev RA Glushtsov AI. Two types of cytotoxic lymphocyte regulation explain kinetics of immune response to human immunodeficiency virus. Proc Natl Acad Sci U S A (2006) 103(3):666–71. 10.1073/pnas.0510016103 16407101 PMC1334670

[54] Wei X Ghosh SK Taylor ME Johnson VA Emini EA Deutsch P Viral dynamics in human immunodeficiency virus type 1 infection. Nature (1995) 373(6510):117–22 10.1038/373117a0 7529365

[55] Ho DD Neumann AU Perelson AS Chen W Leonard JM Markowitz M. Rapid turnover of plasma virions and CD4 lymphocytes in HIV-1 infection. Nature (1995) 373(6510):123–6. 10.1038/373123a0 7816094

[56] Wick WD Yang OO Corey L Self SG. How many human immunodeficiency virus type 1-infected target cells can a cytotoxic T-lymphocyte kill? J Virol (2005) 79(21):13579–86. 10.1128/JVI.79.21.13579-13586.2005 16227278 PMC1262579

[57] Jetzt AE Yu H Klarmann GJ Ron Y Preston BD Dougherty JP. High rate of recombination throughout the human immunodeficiency virus type 1 genome. J Virol (2000) 74(3):1234–40. 10.1128/JVI.74.3.1234-1240.2000 10627533 PMC111457

[58] Zhuang J Jetzt AE Sun G Yu H Klarmann G Ron Y Human immunodeficiency virus type 1 recombination: rate, fidelity, and putative hot spots. J Virol (2002) 76(22):11273–82. 10.1128/JVI.76.22.11273-11282.2002 12388687 PMC136766

[59] Shriner D Rodrigo AG Nickle DC Mullins JI. Pervasive genomic recombination of HIV-1 in vivo. Genetics (2004) 167(4):1573–83. 10.1534/genetics.103.023382 15342499 PMC1470992

[60] Kouyos RD Althaus CL Bonhoeffer S. Stochastic or deterministic: what is the effective population size of HIV-1? Trends Microbiol (2006) 14(12):507–11. 10.1016/j.tim.2006.10.001 17049239

[61] Ribeiro R Bonhoeffer S Nowak M. The frequency of resistant mutant virus before antiviral therapy. AIDS (1998) 12(5):461. 10.1097/00002030-199805000-00006 9543443

[62] Nowak MA Lloyd AL Vasquez GM Wiltrout TA Wahl LM Bischofberger N Viral dynamics of primary viremia and antiretroviral therapy in simian immunodeficiency virus infection. J Virol (1997) 71(10):7518–25. 9311831 10.1128/jvi.71.10.7518-7525.1997 PMC192098

[63] Little SJ McLean AR Spina CA Richman DD Havlir DV Viral dynamics of acute HIV-1 infection. J Exp Med (1999) 190(6):841–50 10.1084/jem.190.6.841 10499922 PMC2195636

[64] Chun T-W Carruth L Finzi D Shen X DiGiuseppe JA Taylor H Quantification of latent tissue reservoirs and total body viral load in HIV-1 infection. Nature (1997) 387:183–8. 9144289 10.1038/387183a0

[65] Kinloch-de Loës S Hirschel BJ Hoen B Cooper DA Tindall B Carr A A controlled trial of zidovudine in primary human immunodeficiency virus infection. N Engl J Med (1995) 333(7):408–13. 10.1056/NEJM199508173330702 7616989

[66] Ho DD Viral counts count in HIV infection. Science (1996) 272(5265):1124–5 10.1126/science.272.5265.1124 8638155

[67] McMichael AJ Borrow P Tomaras GD Goonetilleke N Haynes BF. The immune response during acute HIV-1 infection: clues for vaccine development. Nat Rev Immunol (2010) 10(1):11–23. 10.1038/nri2674 20010788 PMC3119211

[68] Tsimring LS Levine H Kessler DA RNA virus evolution via a fitness- space model. Phys Rev Lett (1996) 76(23):4440–3 10.1103/PhysRevLett.76.4440 10061290

[69] Rouzine IM Coffin JM. Evolution of human immunodeficiency virus under selection and weak recombination. Genetics (2005) 170(1):7–18. 10.1534/genetics.104.029926 15744057 PMC1449738

[70] Achaz G Palmer S Kearney M Maldarelli F Mellors J Coffin J A robust measure of HIV-1 population turnover within chronically infected individuals. Mol Biol Evol (2004) 21(10):1902–12. 10.1093/molbev/msh196 15215321

[71] Brown AJL. Analysis of HIV-1 env gene sequences reveals evidence for a low effective number in the viral population. Proc Natl Acad Sci U S A (1997) 94(5):1862–5. 10.1073/pnas.94.5.1862 9050870 PMC20008

[72] De Boer RJ Oprea M Antia R Murali-Krishna K Ahmed R Perelson AS. Recruitment times, proliferation, and apoptosis rates during the CD8+ T-cell response to lymphocytic choriomeningitis virus. J Virol (2001) 75(22):10663–9. 10.1128/JVI.75.22.10663-10669.2001 11602708 PMC114648

[73] Barton NH Linkage and the limits to natural selection. Genetics (1995) 140(2):821–41. 7498757 10.1093/genetics/140.2.821 PMC1206655

[74] Mueller JC. Linkage disequilibrium for different scales and applications. Brief Bioinform (2004) 5(4):355–64. 10.1093/bib/5.4.355 15606972

[75] Slatkin M. On treating the chromosome as the unit of selection. Genetics (1972) 72(1):157–68. 4672513 10.1093/genetics/72.1.157 PMC1212809

[76] Gorelick R Laubichler M. Decomposing multilocus linkage disequilibrium. Genetics (2004) 166(3):1581–3. 10.1534/genetics.166.3.1581 15082571 PMC1470787

[77] Boutwell CL Rolland MM Herbeck JT Mullins JI Allen TM. Viral evolution and escape during acute HIV-1 infection. J Infect Dis (2010) 202(Suppl 2):S309. 10.1086/655653 20846038 PMC2945609

[78] Fischer W Ganusov VV Giorgi EE Hraber PT Keele BF Leitner T Transmission of single HIV-1 genomes and dynamics of early immune escape revealed by ultra-deep sequencing. PLoS One (2010) 5(8):e12303. 10.1371/journal.pone.0012303 20808830 PMC2924888

[79] Leviyang S. Computational inference methods for selective sweeps arising in acute HIV infection. Genetics (2013) 194(3):737–52. 10.1534/genetics.113.150862 23666940 PMC3697977

[80] O’Connor S Becker E Weinfurter J Chin E Budde M Gostick E Conditional CD8+ T cell escape during acute simian immunodeficiency virus infection. J Virol (2012) 86(1):605–9. 10.1128/JVI.05511-11 22013056 PMC3255930

[81] da Silva J. The dynamics of HIV-1 adaptation in early infection. Genetics (2012) 190(3):1087–99. 10.1534/genetics.111.136366 22209906 PMC3296244

[82] Kessinger TA Perelson AS Neher RA. Inferring HIV escape rates from multi-locus genotype data. Front Immunol (2013) 4:252. 10.3389/fimmu.2013.00252 24027569 PMC3760075

[83] Zagordi O Bhattacharya A Eriksson N Beerenwinkel N. ShoRAH: estimating the genetic diversity of a mixed sample from next-generation sequencing data. BMC Bioinformatics (2011) 12:119. 10.1186/1471-2105-12-119 21521499 PMC3113935

[84] Prosperi MC Salemi M. QuRe: software for viral quasispecies reconstruction from next-generation sequencing data. Bioinformatics (2012) 28(1):132–3. 10.1093/bioinformatics/btr627 22088846 PMC3244773

