# Supplementary material for: The Effect of Interference on the CD8+ T Cell Escape Rates in HIV
Source: Front Immunol. 2015 Jan 13;5:661. doi: 10.3389/fimmu.2014.00661 (PMC4292734; doi:10.3389/fimmu.2014.00661)
Supplement: Supplementary file 1 [file Presentation_1.PDF]

Frontiers in Immunology

Research Article
10 December 2014

Supplementary Material: The effect of interference on the $\mathrm{CD8}^+$ T cell escape rates in HIV

Victor Garcia [1,*], **Roland R. Regoes** [1]

[1] *Institute of Integrative Biology, Department of Environmental Systems Science, ETH Zürich, Zurich, Switzerland*

Correspondence*:
Victor Garcia
Institute of Integrative Biology, Department of Environmental Systems Science, ETH Zurich, Zurich, Switzerland, victor.garcia@env.ethz.ch

MOTIVATION AND EXPLORATION OF CUMULATIVE LINKAGE DISEQULIBRIUM AS PROXY FOR EXPRESSED INTERFERENCE IN HAPLOTYPE DYNAMICS

Here, we motivate the use of cumulative linkage disequilibrium as a proxy for the expressed interference in a haplotype or strain dynamics.

BEHAVIOR OF CUMULATIVE LD

In models of asexual evolution, different regimes such as the sequential fixation regime or the concurrent mutations regime Gerrish and Lenski (1998); Desai and Fisher (2007) do have one common property: Adaptation is characterized by the sequential accumulation of beneficial mutations. The population is thought to evolve as a traveling wave in a one-dimensional fitness-space Tsimring et al. (1996); Rouzine et al. (2003); Rouzine and Coffin (2005); Desai and Fisher (2007). The mean number of mutations in the population increases linearly with time, each sweep of an additional beneficial mutation moving the wave forward towards higher fitness.

However, the composition of the strains that sweep through the population when a beneficial mutation is being acquired is distinct between these regimes. In the sequential fixations regime, a sweep is always composed by one single strain Desai and Fisher (2007). For example, in a two-locus two allele system with the strains ab, Ab, aB, AB, where the caption-alleles are assumed to confer a positive selective advantage, the sequential fixations would involve only one single-mutant haplotype per sweep: $ab \rightarrow aB \rightarrow AB$. On the other hand, in the concurrent mutations regime, the intermediate sweep could be composed by distinct strains with one beneficial mutation each $ab \rightarrow aB, Ab \rightarrow AB$. We think of interference between beneficial mutations in such a simplified system in the latter way, namely by the coexistence of strains with similar selective advantages conferred by different mutations at distinct loci.

We distinguish between the interference that is *expressed* in a given time course of haplotype strains – which may vary from instance to instance due to its stochastic nature – and the interference characterized by the parameter set governing the dynamics. For example, a particular parametrization of a HIV-dynamics model might make the visible and detectable coexistence of similarly fit strains more likely. However, such a signature might not be present in one particular time course instance generated by one single simulation run or in one single patient. On average though, everything else being equal, an appropriate measure for interference should on average be captured over repeated simulation runs. Cumulative linkage disequilibrium is expected to detect the expressed interference in one single

simulation, but not whether the underlying process generating that instance of coexistence is likely to produce similar dynamics in distinct instances. To do the second, repeated simulations are required.

The reasons why cumulative linkage disequilibrium can capture the effect of interference on escape rate decrease are twofold. According to the simplified picture of adaptive evolution described above, the haplotype dynamics can be characterized by two basic parameters. First, the time span between the selective sweeps. Second, the relative frequencies of the intermediate strains with one mutation only, i. e. the single mutants. We show conceptually why on the average, under the variation of these two parameters, the cumulative linkage disequilibrium tends to become larger as the escape rate decrease measures becomes larger.

To do this, we generated artificial non-stochastic escape dynamics in the following way: First, the wildtype is replaced by a composite of two fitter strains, each of which has the same selective advantage compared to the wildtype. The sum of the two fitter strains $Ab + aB$ is going to fixation as described by logistic model $f(t) = f_0/(f_0 - (1 - f_0)e^{-\epsilon t})$, where f_0 is the initial relative frequency of the sum of the single mutants with respect to the wildtype and ϵ is the escape rate Ganusov et al. (2011); Asquith et al. (2006). Here, the logistic function for the fixation is each time determined by an escape rate $\epsilon = 0.3$ and an initial frequency of $f_0 = 10^{-5}$. After a pre-determined time span, the intermediate strains are themselves replaced by the full escape variant AB. The relative frequency of the intermediate strains with respect to one another is also predetermined. Additionally, for each situation considered we sample six times at equally-spaced intervals. We then fit a logistic escape model Asquith et al. (2006); Ganusov et al. (2011) to the sampled data of the frequencies of the beneficial mutants A and B in the population. These fits provide estimates for the escape rates and escape times. With these preparations, we can investigate the behavior of cumulative linkage disequilibrium as well as escape rate decrease with respect to variations in these parameters.

Figure S1 shows how the measure of cumulative linkage disequilibrium changes under the variation of time spans between selective sweeps. In the top row it is shown that the relative frequencies (1:9) between the single mutant strains are the same in both haplotype dynamics. The color coding here is identical to one in the main manuscript, where each color at a specific time point corresponds to the population frequency of a haplotype. Hence, the minimal LD attained in both scenarios is equal, but the cumulative LD differs (second row). The third row shows the fits of a logistic model of escape to the escapes of alleles A and B. In the fourth row, the effect of the time spans on escape rate decrease is shown. The two distinct columns A) and B) show how the difference in the time spans between the first and second sweep translate into the relevant measures of our study. In the first column A), the time to the second sweep is 20 days, in the second column B) it is 50 days.

We first observe that under the assumption of neutrality between the intermediate single mutant strains, the cumulative linkage disequilibrium must roughly scale as the coexistence time of the intermediate strains. Furthermore, with increasing cumulative linkage disequilibrium measures, additional sample points are more likely to come to lie into the coexistence time of the single mutant strains. Therefore, the detection probability for a a situation with interference also increases with increasing cumulative linkage disequilibrium. The additional sample points within the intermediate period of the dynamics are likely to decrease the inferred escape rate of the second allele with respect to the first. This is due to minimization of the difference between the fit curve to the intermediate sample points, which makes the logistic escape function flatter. As can be observed in the last row of the graph, the first escape is almost identical in both situations, but the second escape occurs later and is slower in the second column.

Figure S2 shows how cumulative LD is affected by changing single mutant frequencies. In one extreme, shown in the left column A), the intermediate phase is strongly dominated by one of the strains. This situation is only little different from a pure sequential fixation dynamics. Correspondingly, the signal for cumulative linkage disequilibrium is small, as well as the escape rate decrease. As the relative frequency of the intermediate strains becomes more balanced (as shown in the middle column B)), a stronger signal for cumulative LD is the consequence. At the same time, the fit for the first allele going to fixation will be flatter than in the previous case with almost no cumulative linkage disequilibrium. This is because the

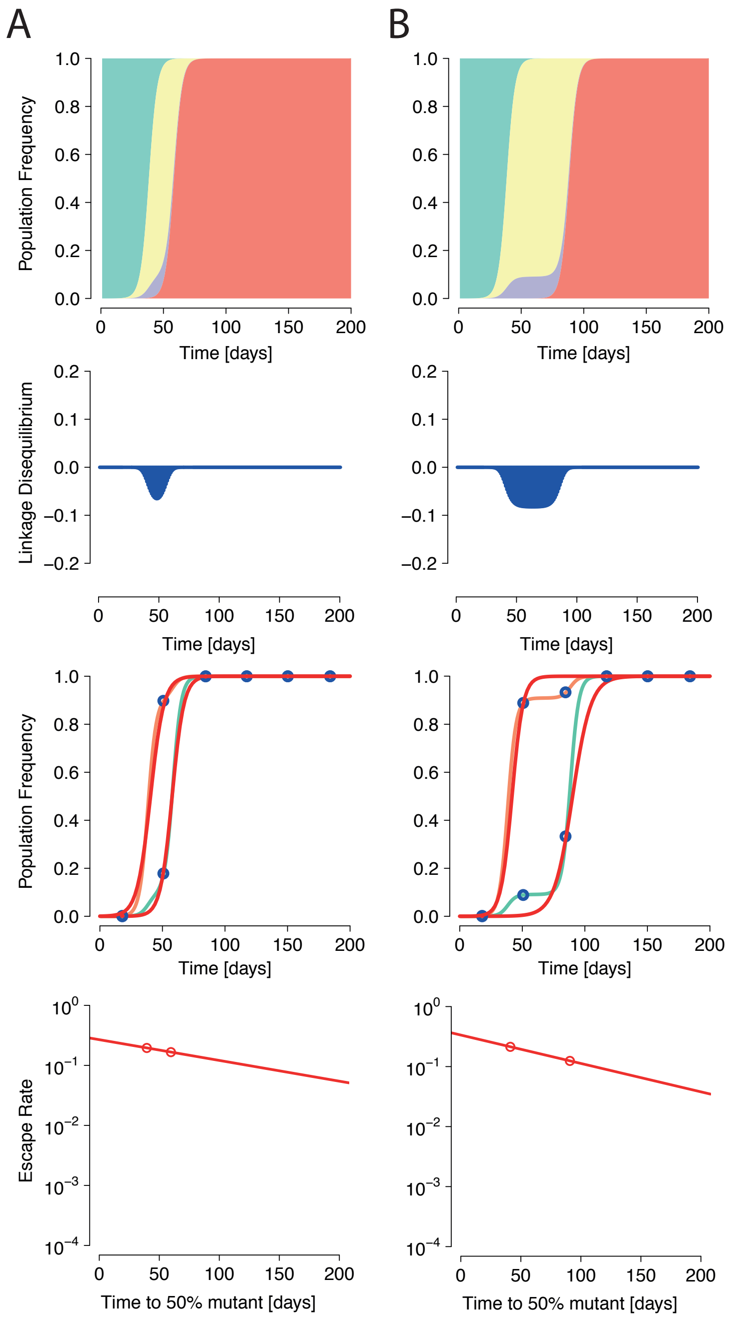

Figure S1. Behavior of cumulative linkage disequilibrium with increasing time spans between escapes and equal relative frequencies of single mutants. The two distinct columns A) and B) show how the difference in the time spans between the first sweep of single mutants and second sweep of double mutants impact LD and escape rate decrease. In the first column A), the time to the second sweep is 20 days, in the second column B) it is 50 days. The top row shows that the relative frequencies (1:9) between the single mutant strains are the identical in both haplotype dynamics. The population frequency of the wildtype ab is shown in green, the frequencies of the single mutants Ab and aB are shown in yellow and violet respectively, and the double mutant (AB) frequency is shown in red. The second row shows that the minimal LD attained is the same, but the cumulative LD differs. The third row shows the fits of a logistic model (red) to the sampled escape mutation frequencies of A and B. In the fourth row the escape rates ϵ and escape times τ_{50} inferred from each escape by the logistic model fit are represented as red points. Larger cumulative LD coincide with more negative escape rate decrease slopes.

sample points for the first allele within the intermediate single mutant phase will lie at lower frequencies, and hence the fits will correct by reducing the escape rate parameter. Similarly, the escape rate of the second allele will be reduced. But since the sampling points in the intermediate phase will have low frequency first, this will result in an even flatter fit compared to the first allele. This entails escape rate decrease.

However, for perfect equality in proportions between the single mutant strains (Figure S2C)), the predictive capacity of the cumulative LD breaks down. This is due to the fact that as the strain frequencies become more similar, the allele trajectories become more similar as well. Therefore, there will be little difference between the inferred escape rate times and escape rates. Hence, the escape rate decrease could potentially become infinite. In a more realistic setup, the escape rate decrease will be highly sensitive to stochastic effects, leading to very large variation in estimated values.

This last situation is a major disadvantage of the measure of cumulative linkage disequilibrium. However, in stochastic simulations it is very improbable to be encountered often enough as to have a disrupting effect. As the vast majority of simulation outcomes lie between the situation depicted in the left column and the middle column of figure S2, we can safely neglect this case. We also expect these effects to act on average in the same manner if the sampling rates are such that essential features can still be captured. This is confirmed in the simulations run in the paper.

A TEST OF CUMULATIVE LD FOR A TWO-LOCUS TWO-ALLELE WRIGHT-FISHER MODEL

To prove that cumulative linkage disequilibrium does also (on average) capture interference in better understood systems, we conducted simulation experiments for a Wright-Fisher model. In classical population genetics the transitions from a sequential fixation regime into a concurrent mutations regime is well understood. We simulated the behavior of the cumulative LD in both regimes and observed how it changed during the transition. As expected, cumulative LD showed a marked increase during the transition. This shows that the emergence of cumulative linkage disequilibrium matches the predictions in classical population genetics, and might therefore be used with the necessary caution in other contexts.

In their seminal paper about clonal interference, Gerrish and Lenski derived a condition under which interference should be expected Gerrish and Lenski (1998). Whether the adaptive evolution of a population is going to be impacted by interference depends on the values of three major parameters, which characterize the behavior of the system. These are the population size N, the beneficial mutation rate per generation and per individual (or sequence) μ_b and the fitness advantage associated with such a beneficial mutation s. Given these parameters, the system is going to be likely to display interference under the following condition:

$$N\mu_b \gtrsim \frac{1}{\ln Ns}. \tag{1}$$

For fixed μ_b and s, the inequality is eventually going to be satisfied with increasing N. Desai and Fisher Desai and Fisher (2007) refer to this as a transition from the sequential fixations regime into a concurrent mutations regime. We hypothesized that if cumulative linkage disequilibrium was a reasonable measure for interference, it would capture this transition. Therefore, we gradually increased N in simulations of a Wright-Fisher model, and investigated whether negative cumulative linkage disequilibrium would start to appear as the threshold to the concurrent mutations regime is surpassed.

In order to address this question, we implemented a simple Wright-Fisher model with mutation and selection. There exist four different strain types in this model: $\{ab, Ab, aB, AB\}$, corresponding to the possible strains in a two-locus two-allele system. Each of the strains **i** is assigned a fitness, w_i. The fitness is determined by the number of escape mutations in **i**. The wildtype strain ab, has fitness 1, $w_{ab} = 1$. Escape mutations are denoted by capitalized letters, A and B. For each additional escape mutation, it is assumed that an additive selective advantage $s = 0.05$ is conferred to the strain, such that $w_{Ab} = w_{aB} =$

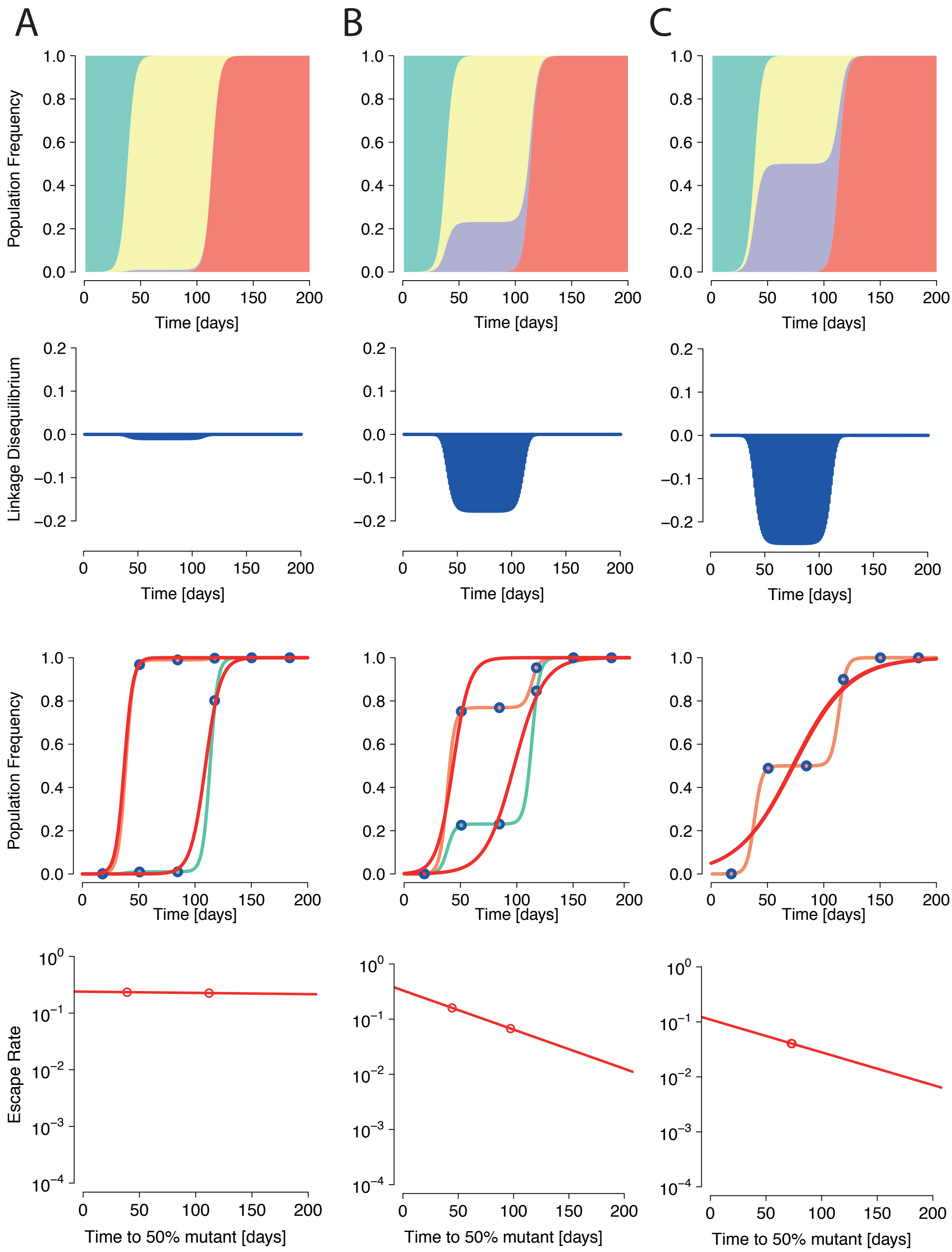

Figure S2. Behavior of cumulative linkage disequilibrium with increasing similarity in single mutant haplotype frequencies. Columns A) to C) show the impact of more and more similar relative single mutant frequencies on the cumulative LD and the escape rate decrease. Column A) shows the effects of relative frequencies 1:100, column B) of relative frequencies 1:4 and column C) of relative frequencies 1:1 between single mutants. The time span between sweeps is held fixed in all columns. The rows are as in Figure S1.

$1+s$ and $w_{AB} = 1+2s$. Mutations $a \to A$ and $b \to B$ occur at a rate $\mu_b = 10^{-4}$ per strain per generation. No backward mutations are considered. The beneficial mutation rate μ_b in the model was assumed to be 1×10^{-4}.

Simulations are initiated with a homogeneous population of wild-type strains. For each of the values of $N = \{10^2, 5 \times 10^2, 10^3, 5 \times 10^3, 10^4, 5 \times 10^4, 10^5\}$, we repeated the simulations 100 times, up to 2000 generation steps. After each generation step, the population was resampled from the previous generation. The new generation is drawn from a multinomial distribution, in which the probability for a strain **i** to be drawn is given by $p_i = \frac{n_i}{N} \times w_i/\overline{w}$, where $\overline{w} = \sum_i w_i \frac{n_i}{N}$ is the average fitness and n_i is the number of individuals of type **i** in the past generation.

Figure S3 shows the distribution of the cumulative LD measured in each simulation across the parameter space of N considered. The transition point from sequential fixations regime to concurrent mutations regime N_0 is at 2140. In the figure, we see a sharp decay in the median cumulative LD into negative values after this transition point has been trespassed. These negative values are maintained over at least two orders of magnitude. They are expected to disappear as soon as $N_e \mu_b^2 \approx 1$, where double mutants start to emerge almost immediately. In this regime, the cumulative LD turns positive.

We conclude that for the population sizes of interest, negative cumulative linkage disequilibrium produces a consistent signal when the interference regime is reached. The statistic is therefore well-behaved in the well-studied Wright-Fisher model. Thus, it is legitimate to explore its applicability in more complex model environments.

CALCULATION OF FORWARD AND REVERSE MUTATION RATES

Cells infected with strain **x** that are in the compartment $I_{\mathbf{x}}$ mutate into cells infected with a distinct strain, **y**, at rate $m_{\mathbf{xy}}$. Since strains are represented by a string of binary digits, which represent mutations at a particular epitope-coding locus, there exist two possible types of mutations. Forward mutations change zeroes in a locus to ones at rate μ_f, and reverse mutations do the opposite at rate μ_b. We generally assume that an epitope consists of about $m = 8$ codons. Each nucleotide is mutated with a probability of $3 \cdot 10^{-5}$ per replication Mansky and Temin (1995).

The probability calculations for an escape epitope to emerge μ_f are based on the assumption that escapes are the consequences of a change to any of the coding nucleotides within a codon. The mutation probability per replication per base pair is μ. The probability not to alter the codon amounts to the probability not to alter any base pair in the triplet or alternatively, only to alter a non-coding base pair. This probability is given by: $p_n = (1-\mu)^3 + \mu \cdot (1-\mu)^2 \cdot 0.78$. The factor 0.78 arises from the fact that modifying the last base pair of the codon leads to a change in the amino-acid in about 22 percent of cases (under the assumption that all base pairs are equally probable replacement candidates). Hence the probability to *alter* the triplet is one minus that quantity, leading to codon altering rate $\mu_c = 1 - p_n$. Analogously, the probability not to hit any triplet is given by $(1-\mu_c)^m$. Again, one minus that quantity is the probability to alter some triplet in the epitope sequence. Therefore the probability for a zero at position j in a strain **x** to change into a 1 at the same position (representing a mutation modifying an epitope) is $\mu_f = (1 - (1-\mu_c)^m) \cdot 0.2$. Here, the factor 0.2 accounts for the fact that only a fraction of the mutants is non-lethal or deleterious, and has a mutation enabling it to escape immune responses. In Lalić et al. (2011); Sanjuán et al. (2004), it is shown that about 50% of mutations are completely deleterious. Out of the remaining 50% deleterious to beneficial mutations, we assume that about half are in an intermediate range of deleteriousness, not being immediately cleared by purifying selection. This estimate was set to be large in order to make conservative assumptions for the simulations. With these assumptions, we obtain $\mu_f \approx 10^{-4}$.

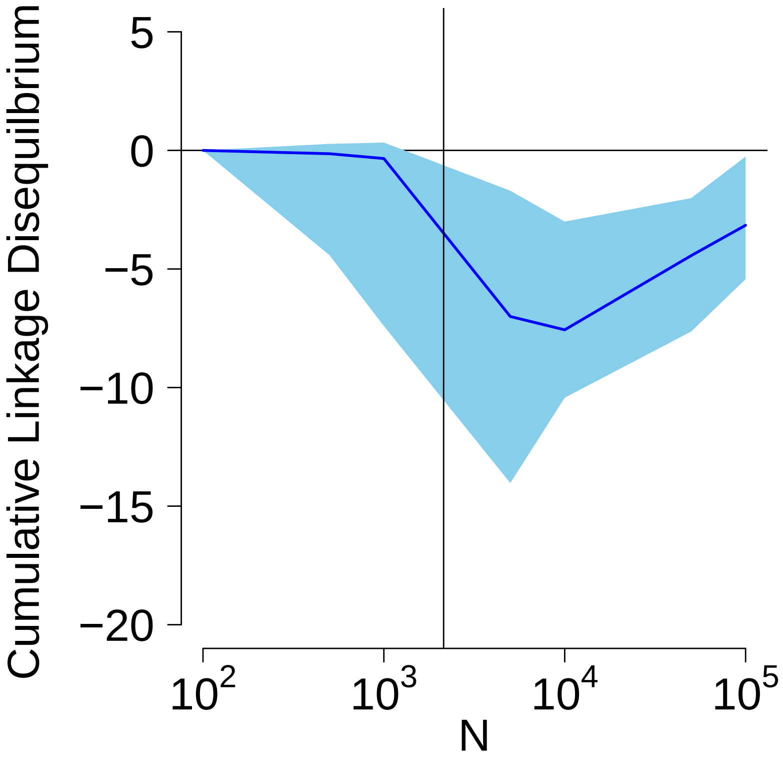

Figure S3. Behavior of cumulative linkage disequilibrium as a system transitions from the sequential fixations regime to the multiple mutations regime. The variation in cumulative linkage disequilibrium values in 100 simulation repeats is plotted against distinct values of N. The dark blue line is the median of 100 simulations, the upper and lower end of the blue-shaded area are the 95 and 5 percentiles of all measured simulation runs, respectively. The median cumulative linkage disequilibrium becomes negative as the transition point dividing the sequential fixations regime and the concurrent mutations regime is trespassed. A vertical black line goes through $N_0 = 2140$.

For the reverse rate μ_b, namely for converting a 1 at particular position of a modeled strain **x** to a zero, it is necessary to alter exactly that nucleotide which has been changed when converted from a zero to a one. This probability corresponds to $\mu_b = \mu \cdot \frac{1}{m} \cdot (1/2) \cdot (1/3)$, which is the probability to mutate and to hit the right triplet of nucleotides in the sequence, times the probability to hit the right coding nucleotide, times the probability to attain the correct back-mutation (one out of three possible nucleotides). This value amount to 6.25×10^{-7} with $\mu = 3 \cdot 10^{-5}$, and to 4.48×10^{-7} with $\mu = 2.15 \cdot 10^{-5}$, which is a better estimate for the point mutation rate in HIV. We therefore chose $\mu_b = 5 \times 10^{-7}$ for simplicity.

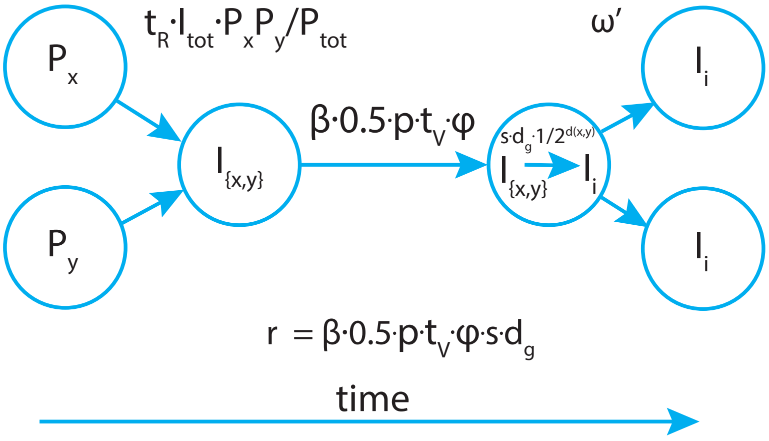

Figure S4. Diagrammatic representation of the modeled recombination process. Virions carrying *parent strains* from productively infected cells infect a target cell. The newly infected cell co-packages both strains and reinfects a new cell. In the new cell, the two strains recombine into a new *offspring* strain. The rates or factors for the processes are given above the arrows.

DETAILED DERIVATION OF RECOMBINATION TERM

$$\frac{d}{dt}I_{\mathbf{i}} \propto r \cdot t_R \cdot I_{tot}\left(\sum_{\mathbf{x,y}\in Q(\mathbf{i})}\frac{P_{\mathbf{x}}P_{\mathbf{y}}}{P_{\text{tot}}^2}\frac{1}{2^{d(\mathbf{x,y})}}\omega'(\mathbf{x,y,i}) - \sum_{\mathbf{x}}\frac{2P_{\mathbf{x}}P_{\mathbf{i}}}{P_{\text{tot}}^2}\frac{2^{d(\mathbf{x,i})}-1}{2^{d(\mathbf{x,i})}}(-\omega'(\mathbf{x,i,i}))\right) \tag{2}$$

(3)

Equation 2 describes how the change in infected cells is affected by recombination. *Parent strains* **x** and **y** which co-occur in a cell can recombine into new strains **i**. Equally, when **i** inserts itself into a cell already infected with another strain, it might recombine into a distinct strain and therefore be lost.

In order to analyze these processes, we first calculate the number of infected cells of type **i** that are added to the compartment $I_{\mathbf{i}}$ per day and stem from two recombining strains **x** and **y**. This approach is similar to the one in Mostowy et al. (2011). Schematically, this process is represented in Figure S4. First, we note that a population of doubly infected cells $t_R \times I_{\text{tot}}$ is maintained at all times. The fraction of multiply infected cells is set to $t_R = 5 \cdot 10^{-3}$, in accordance with Jung et al. (2002); Josefsson et al. (2010); Neher and Leitner (2010); Batorsky et al. (2011); Mostowy et al. (2011). A fraction $\frac{P_{\mathbf{x}}P_{\mathbf{y}}}{P_{\text{tot}}^2}$ of this population has been infected with both of the relevant parent strains.

Out of these appropriately co-infected cells, a fraction of $\frac{1}{2}$ of cells will produce co-packaged virions with both strains. Each of the co-infected cells produces about p virions in its life-span of a day. β is the maximum rate at which a virion infects a new cell before being cleared or damaged otherwise. Therefore, β is equivalent to the maximum rate of newly infected cells per virion De Boer (2007). Hence, the number of newly infected cells stemming from a single doubly infected cell is going to $\beta \cdot p \cdot t_V \cdot \frac{1}{2} \cdot \phi$, where $\phi = \langle \frac{T}{h_\beta + T} \rangle$ is a correction factor to account for the reduced infectivity of a virion for most of the HIV-infection. Therefore, $tI_{\text{tot}}\frac{P_{\mathbf{x}}P_{\mathbf{y}}}{P_{\text{tot}}^2}\beta p t_V \frac{1}{2}\phi$ is the number of cells infected with virions co-packaged with **x** and **y** maintained in the population.

In these cells, the co-packaged strains are recombined after introduction. We assume the template switching rate to be s and the crossover events to be uniformly distributed across the genome. Furthermore, we assume all the beneficial escape mutations to be located within some range d_g (measured in bp units) in the HIV-genome with respect to one another. Hence, a crossover event is going to occur within that range roughly with a probability of $d_g \times s$ per generation (that is, per day). This is the rate of production of recombinant strains. $2^{d(\mathbf{x},\mathbf{y})}$ distinct recombinant offspring types can potentially arise out of a pair $(\mathbf{x},\mathbf{y})$ undergoing reverse transcriptase, where $d(\mathbf{x},\mathbf{y})$ denotes the Hamming distance between two strains. Hence, the probability of $\mathbf{i}$ to be the recombinant offspring is $1/2^{d(\mathbf{x},\mathbf{y})}$. As shown in Figure S4, the creation of a strain $\mathbf{i}$ by recombination is preceded by the loss of two strains $\mathbf{x}$ and $\mathbf{y}$. We therefore multiply the output by $\omega'(\mathbf{x},\mathbf{y},\mathbf{i})$ in order compensate for this effect. $\omega'(\mathbf{x},\mathbf{y},\mathbf{i})$ is defined such that it is equal to two if none of the parent strains were identical to the offspring strain, and equal to 1 if one of the parent strains is identical to the offspring strain, and zero if both are identical: $\omega'(\mathbf{x},\mathbf{y},\mathbf{i}) = 2 - \mathbf{1}_{\mathbf{x}=\mathbf{i}} - \mathbf{1}_{\mathbf{y}=\mathbf{i}}$, where $\mathbf{1}_{\mathbf{y}=\mathbf{i}}$ is one if $\mathbf{y} = \mathbf{i}$ and zero otherwise.

Since this calculation applies for one specific pair of parent strains $(\mathbf{x},\mathbf{y})$ only, the total contribution to the infected cells of type $\mathbf{i}$ is given by the sum over all possible parent pairs $Q(\mathbf{i})$.

We also considered the loss to the compartment $I_{\mathbf{i}}$ per day due to recombination. The derivation is completely analogous to the first, where recombination processes increase the number of infected cells, simply replacing one of the parent strains with $\mathbf{i}$.

We define the baseline recombination rate r to be $r = \beta p t_V \frac{1}{2}\phi d_g s$. This a combination of factors which is independent of the strains or their frequencies, which incorporates several distinct rates and probabilities. In our model, we choose $p t_V \frac{1}{2}\phi d_g s \approx 0.5$, with $p \approx 10^4$ Hockett et al. (1999); Reilly et al. (2007); Chen et al. (2007); De Boer et al. (2010), $t_V \approx 0.01$, Ramratnam et al. (1999); De Boer et al. (2010), $\phi \approx 1/100$ (estimated from our simulations), and $s d_g \approx 0.5$, where $s \approx 3 \times 10^{-4}$ per bp per generation Jetzt et al. (2000); Zhuang et al. (2002). This leads to $r \approx 1.4 \times 10^{-4}$.

RECOMBINATION TERM IN MODEL PROPORTIONAL TO LINKAGE DISEQUILIBRIUM

The individual terms for recombination given in the last term of equation 2 in the manuscript encode all possible recombination events between all the strain types modeled. Here, we want to prove that for the case of two loci, this term is effectively proportional to the linkage disequilibrium D between the productively infected cells. Linkage disequilibrium in a population consisting of individuals endowed with two loci, and with two possible alleles at each locus, 0 and 1, is given by:

$$D = p_{00}p_{11} - p_{01}p_{10}, \tag{4}$$

where $p_{\mathbf{i}}$ stands for the relative frequency of the strain type $\mathbf{i}$. In this part, we will utilize the notation $p_{\mathbf{x}} = P_{\mathbf{x}}/P_{\mathrm{tot}}$ for the relative frequency of the number of productively infected cells of strain type $\mathbf{x}$. We calculate the expression given in equation 2 of the manuscript for the reference strain $\mathbf{i} = (00)$, by evaluating each individual expression:

$$I_{\mathbf{i}} \propto rt_R \cdot I_{tot} \left(\sum_{\mathbf{x},\mathbf{y} \in Q(\mathbf{i})} \frac{P_{\mathbf{x}} P_{\mathbf{y}}}{P_{\text{tot}}^2} \frac{1}{2^{d(\mathbf{x},\mathbf{y})}} \omega'(\mathbf{x},\mathbf{y},\mathbf{i}) - \sum_{\mathbf{x}} \frac{2 P_{\mathbf{x}} P_{\mathbf{i}}}{P_{\text{tot}}^2} \frac{2^{d(\mathbf{x},\mathbf{i})} - 1}{2^{d(\mathbf{x},\mathbf{i})}} (-\omega'(\mathbf{x},\mathbf{i},\mathbf{i})) \right) = \tag{5}$$

$$\begin{aligned} rt_R \cdot I_{tot} \cdot (0 \cdot 1 \cdot p_{00}^2 + \frac{1}{2} \cdot 1 \cdot p_{00} p_{01} + \frac{1}{2} \cdot 1 \cdot p_{00} p_{10} + \frac{1}{4} \cdot 1 \cdot p_{00} p_{11} \\ + \frac{1}{2} \cdot 1 \cdot p_{01} p_{00} + \frac{1}{4} \cdot 2 \cdot p_{01} p_{10} + \frac{1}{2} \cdot 1 \cdot p_{10} p_{00} + \frac{1}{4} \cdot 2 \cdot p_{10} p_{01} + \frac{1}{4} \cdot 1 \cdot p_{11} p_{00} \\ -2(0 \cdot 0 \cdot p_{00}^2 + \frac{1}{2} \cdot 1 \cdot p_{00} p_{01} + \frac{1}{2} \cdot 1 \cdot p_{00} p_{10} + \frac{3}{4} \cdot 1 \cdot p_{00} p_{11})) = \end{aligned} \tag{6}$$

$$\begin{aligned} rt_R \cdot I_{\text{tot}} \cdot (p_{10} p_{01} + (p_{00} p_{01} - \frac{2}{2} \cdot p_{00} p_{01}) + (p_{00} p_{10} - \frac{2}{2} \cdot p_{00} p_{10}) + (\frac{1}{2} \cdot p_{00} p_{11} - 2 \cdot \frac{3}{4} \cdot p_{00} p_{11})) = \\ rt_R I_{\text{tot}} \left(p_{10} p_{01} - p_{00} p_{11} \right) = -rt_R I_{\text{tot}} D \end{aligned}$$

The calculation for the reference strains (01), (10) and (11) is analogous to the one presented. For strains containing larger loci numbers, the calculations become increasingly complicated.

RESCALING SYSTEM SIZE FOR STOCHASTICITY CONTROL

The stochastic effects in the simulations are quantified by the extent to which stochastic events (commonly encoded by rate terms) alter simulation variables in magnitude. Thus, increasing the stochasticity of a simulation is equivalent to downsizing the entire system simulated. The events simulated by the Gillespie algorithm alter the amounts of the simulation variables in units of one. Therefore, the smaller the total magnitude of a particular simulation variable, the more affected it is by the process noise.

By downsizing or magnifying a system we refer to transforming the system under consideration S, with its variables T, I_i, P_i into a system labeled S_a, which has been scaled by a factor a and has corresponding variables $T_a, I_{i,a}, P_{i,a}$. The deterministic dynamics of the downsized system S_a has the property that each of its variables has the same time course as the corresponding variables in S, multiplied by a factor a:

$$T_a = T \cdot a \tag{7}$$

$$I_{\mathbf{i},a} = I_{\mathbf{i}} \cdot a \tag{8}$$

$$P_{\mathbf{i},a} = P_{\mathbf{i}} \cdot a \tag{9}$$

A magnification of all individual variables by a implies that the time derivatives are a factor a larger as well. Therefore, all terms that contribute to that time derivative have to be magnified by a individually, in order to keep the equations balanced. This is the guiding principle to identify the scaling factors for the parameters of the system.

In the first equation for T, for instance, a transformation to T_a ensues that all the terms are scaled equally. Therefore,

$$\sigma_a = \sigma \cdot a \tag{10}$$
$$d_{T,a} = d_T \tag{11}$$
$$\beta_a = \beta \tag{12}$$
$$h_{\beta,a} = h_\beta \cdot a. \tag{13}$$

The factors for β and h_β where found in the following way: The interaction terms under the modified system need to have the equal form than the term of the old system times a factor a:

$$\frac{\beta_a T_a p_a P_{\mathbf{i},a}}{h_{\beta,a} + T_a} = a \frac{\beta T p P_{\mathbf{i}}}{h_\beta + T} \tag{14}$$

By virtue of eq. 7, we have that:

$$\frac{\beta_a T_a p_a P_{\mathbf{i},a}}{h_{\beta,a} + T_a} = a^{-1} \frac{\beta T_a p P_{\mathbf{i},a}}{h_\beta + T_a/a}, \tag{15}$$

which can be easily attained by scaling h_β with a and leaving the rest of the parameters unchanged under the transformation, which leads to equations 10. Analogously, the rest of the parameters transform like:

$$d_a = d \tag{16}$$
$$\gamma_a = \gamma \tag{17}$$
$$m_{\mathbf{x},\mathbf{y},a} = m_{\mathbf{x},\mathbf{y}} \tag{18}$$
$$r_a = r \tag{19}$$
$$\delta_a = \delta \tag{20}$$
$$k_a = k \tag{21}$$
$$h_{k,a} = h_k \cdot a. \tag{22}$$

ESCAPE DYNAMICS

Commonly, the fixation of the escape mutant within the population is described by a logistic model Fernandez et al. (2005); Asquith et al. (2006); Ganusov et al. (2011); Henn (2012). As a reference model, we resort to Ganusov et al. (2011), which has also been employed in experimental studies Ganusov et al. (2011); Goonetilleke et al. (2009). In that model, the frequency time course of a mutant going to fixation in the viral population $f(t)$ is given by:

$$f(t) = \frac{f_0}{f_0 + (1 - f_0)e^{-\epsilon t}}, \tag{23}$$

where f_0 is the initial frequency of the mutant and ϵ is the average mutant fitness advantage and used as measure for the escape rate of the mutant. Logistic frequency time courses for fixating mutations are a common result in population genetics for strongly selected mutations Ewens (2004). A further quantity

of interest is the time at which the mutant has reached fifty percent frequency in the population, which is given by:

$$\tau_{50} = \frac{1}{\epsilon} \ln \frac{1 - f_0}{f_0}. \tag{24}$$

This quantity is termed the *escape time* of a particular mutant, and ϵ as *escape rate*. Since both are free parameters of the function, they need to be inferred from experimental data.

SAMPLING METHODS AND FITTING

In order to realistically recreate estimation procedures employed in experimental settings, we have based the choice of sampling times on experimental work Goonetilleke et al. (2009). Samples are taken at times of $0, 10, 20, 50, 100, 190, 300$ days post identification of infection (which we assume to be at day 18), roughly as in the experimental setup of Goonetilleke et al. (2009). We added an artificial measurement error to the frequency data by means of a truncated normal distribution Jackson (2011) with $\sigma_{\text{sample}} = 0.01$.

We then fitted equation (23) to the sample frequencies for escape mutation. The escape rate ϵ and escape time τ_{50} are calculated from the fitted parameters. To find the optimal fit through given data points we used the R-function *optim* Team (2012), with the residual sum of squares as a stress function to be minimized. We constrained the parameters to satisfy $\epsilon > 0$ and $0.06 > f_0 > 0$. The optimization was carried out on the logarithm of f_0, $f_l = \ln(f_0)$, since artefacts are very easily produced otherwise.

ESCAPES IN UNSCALED SYSTEMS SHOW NO OR POSITIVE CUMULATIVE LINKAGE DISEQUILIBRIUM

We investigated how differences in the strength and time delay of CD8$^+$ T cell effector functions affect the HIV dynamics by simulating a variety of combinations of CD8$^+$ T cell time courses in unscaled systems ($a = 1$). For each simulation, we combined two CD8$^+$ T cell functions. Each CD8$^+$ T cell function was characterized by one of two different values for the final level, $K_j \approx 1 \cdot 10^7$ or $K_j \approx 7 \cdot 10^6$, where $j \in \{1, 2\}$ denotes the order of elicitation. The first elicited function $j = 1$ is always set to start growing at simulation time $t = 0$. The second $j = 2$ starts with a predetermined time delay. Simulations were run for different time delays between the CD8$^+$ T cell functions. The time delays where chosen from 0 to 30 in steps of five days. Each combination of CD8$^+$ T cell function pairs was simulated 100 times.

To test whether selective interference was present in the simulations, we calculated the cumulative linkage disequilibrium of each simulation. Figure S5 shows the results of the cumulative linkage disequilibrium for increasing, equal and decreasing CD8$^+$ T cell function strengths. The results indicate that selective interference did not appear in any of the configurations tested. This is due to the presence of double mutants in the population upon the onset of the immune responses. If the population size N is given by $N = 10^{10}$, and the effective mutation probability for an epitope into an escape epitope is $\mu = 10^{-4}$, we would expect a stable number of about 10^2 double mutants to be present. This pre-existence of fitter double mutants crucially impacts the dynamics: they will immediately be selected upon elicitation of the immune responses, making the effect of recombination dispensable.

ESCAPE RATE DECREASE IN DOWNSCALED SYSTEM

For each simulation, we calculated the escape rates from the frequency of the escape alleles (disregarding their linkage). This was done by mimicking experimental procedures, where the frequencies of each escape allele were sampled at predetermined time points (densely sampled at the beginning, sparsely

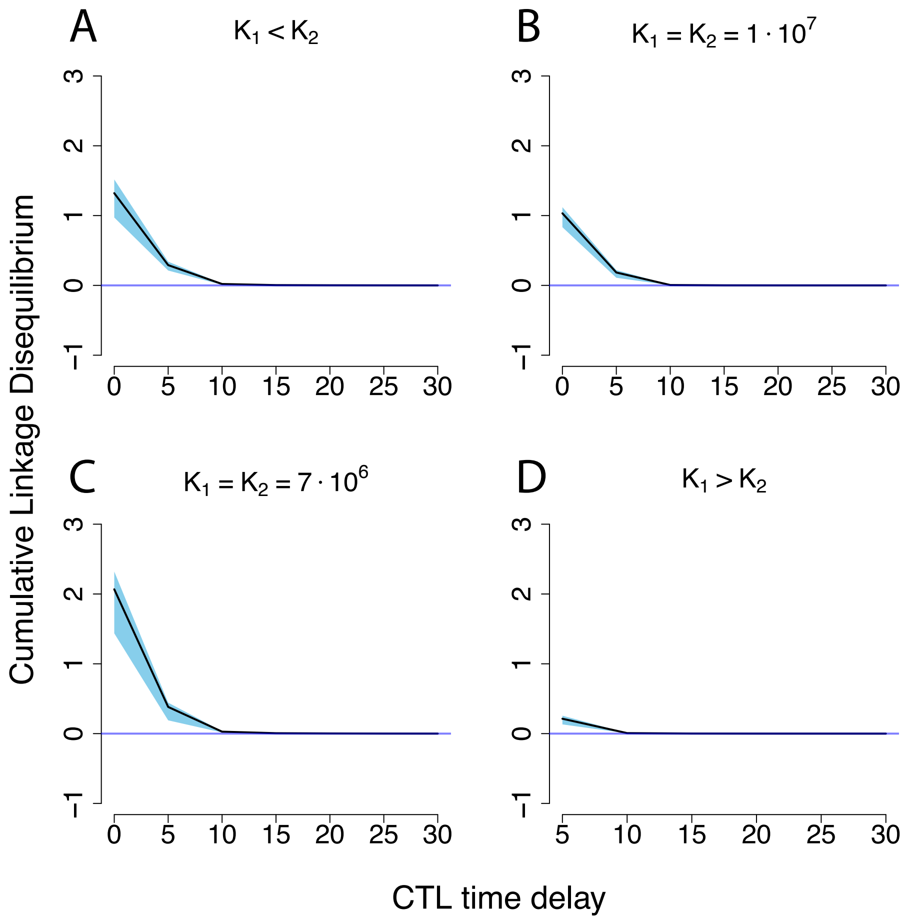

Figure S5. Cumulative Linkage Disequilibrium values for simulation runs differing in CD8$^+$ T cell function strength and timing. The x-axis denotes the time delay the second immune response has to the first. The black line is the median of 100 simulations, the upper and lower end of the blue-shaded area are the 95 and 5 percentiles of all measured simulation runs, respectively. The transparent blue base line is set to zero. K_1 and K_2 denote the settling values for the first and the second immune responses respectively. The cases $K_1 < K_2$ and $K_1 > K_2$ (A) and D)) are nearly identical. Note that the time delay zero simulations are omitted in the figure $K_1 > K_2$, since they are equivalent to those in $K_1 < K_2$. The figures indicate that the further apart the immune responses are elicited, the more likely it is for the escapes to occur sequentially. The closer the CD8$^+$ T cell functions, the higher the chance for a double mutant to be selected. The median cumulative linkage disequilibrium for C), $K_1 = K_2 = 7 \cdot 10^6$, is larger at small delay times than for B), $K_1 = K_2 = 10^7$. This is because smaller settling values of synchronously elicited CD8$^+$ T cell functions select for double mutants whose escape rates are smaller. Therefore the fixation time is larger, and the cumulative linkage disequilibrium value is increased as well.

sampled towards the end of simulation time, as in Goonetilleke et al. (2009); Ganusov et al. (2011) (see *Materials and Methods*). Lastly, these frequency values were then utilized to fit a simple logistic model (see equation (23)), as in Ganusov et al. (2011); Asquith et al. (2006); Henn (2012). The escape time and escape rate for each allele fixation was then calculated from the fitted parameters.

With these values ϵ_1, ϵ_2 and $t_{50,1}, t_{50,2}$, we calculated the successive escape rate decrease in each simulation. This was done by fitting a linear regression on the data pairs $(t_{50,1}, \log_{10}(\epsilon_1))$ and $(t_{50,2}, \log_{10}(\epsilon_2))$, as in Ganusov et al. (2011). Note that the values $\log_{10}(\epsilon)$ do not have a well-defined unit. The slope of the regression, termed *escape rate decrease value* (ERD value) is the value of b from the regression-formula $log_{10}(\epsilon) = a + b \cdot t_{50}$. Escape rate decrease is used as a proxy of successive escape rate change. Negative escape rate decrease indicates that later escapes are slower.

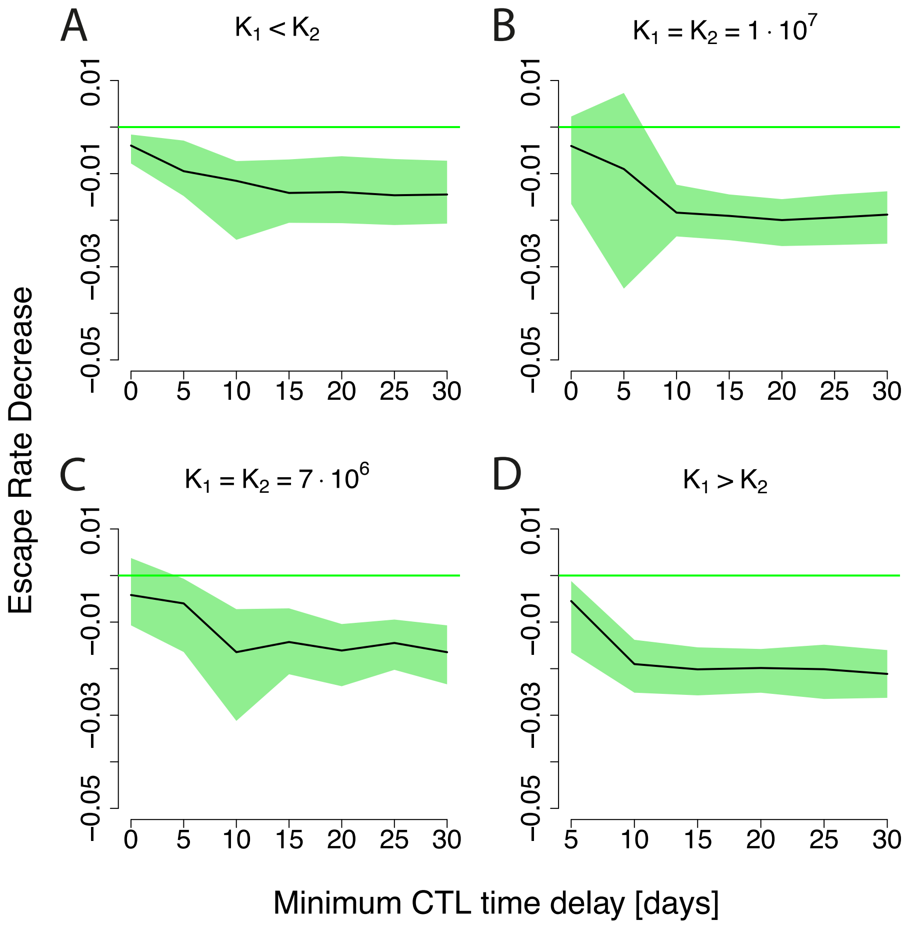

Figure S6. Escape rate decrease values for simulation runs of a scaled down two-locus system (scaling factor $a = 10^{-4}$) differing in CD8^+ T cell function strength and timing. The x-axis denotes the time delay the second immune response has to the first. The black line is the median of 100 simulations, the lower and upper end of the green-shaded area are the 15 and 85 percentiles of all measured simulation runs, respectively. The transparent horizontal green base line goes through zero. K_1 and K_2 are the settling values for the first and the second immune responses, respectively. The medians of figures A), B), C) and D) all increase towards smaller time delay values, where they approach values observed in experiments Ganusov et al. (2011). The zero time delay value in D) is intentionally missing, since this is an identical situation to A).

Figure S6 shows the escape rate decrease values obtained from simulations of a system with high stochasticity ($a = 10^{-4}$). In all of them the median grows consistently larger towards smaller time delays. The median values for all situations remain stable at about -0.02 for time delays larger than 10 days. For small time delays, the escape rate decrease values are centered between -0.01 and 0.

Values of that magnitude entail that escape rates of the order of $0.2\,\mathrm{day}^{-1}$ at around day 15 (after detection of infection) decline an order of magnitude to about $0.01\,\mathrm{day}^{-1}$ at day 200. The difference between the 15 and 85 percent quantiles of the escape rate decrease values remains relatively stable across time delays.

INCREASING DELAY TIMES BETWEEN CD8^+ T CELL RESPONSES LEAD TO GRADUAL REPLACEMENT OF INTERFERENCE PATTERN WITH SEQUENTIAL ESCAPE

In order to control for the result found for synchronously elicited and equally strong CD8^+ T cell responses, we reproduced the same density plot as shown in Figure 7 in the manuscript in five additional cases of substantial negative cumulative linkage disequilibrium. In each case, 1000 simulation runs were performed, but only the non-positive cumulative LD outcomes are represented. These cases indicate a dissociation of interference and escape rate decrease with increasing time delay between the elicitation of the immune responses.

Figure S7 shows the association for equally strong and synchronously elicited immune responses (cases A and C), where the association is more pronounced for C. This is due to the lower CD8^+ T cell numbers in C. Since weaker selective pressures induce a longer coexistence of competing single-mutant strains, this leads to an increased cumulative linkage disequilibrium. Departures from this CD8^+ T cell-response combination by an increase in time delay lead to the appearance of a second density peak at about zero cumulative linkage disequilibrium and negative escape rate decrease values (see cases B, D and E). This peak becomes more pronounced as the time delay between the responses increases (see cases C, D and E, in that order, as well as A and B in that order). Case F has been addressed in the paper and deviates from to the general patterns observed in the rest of the cases due to a particular choice in the timing of CD8^+ T cell response elicitation. In this last case, we identify the coexistence of two modes of sequential escapes either carrying the signature of interference (with large cumulative linkage disequilibrium values) or low cumulative LD values, indicating successive escapes.

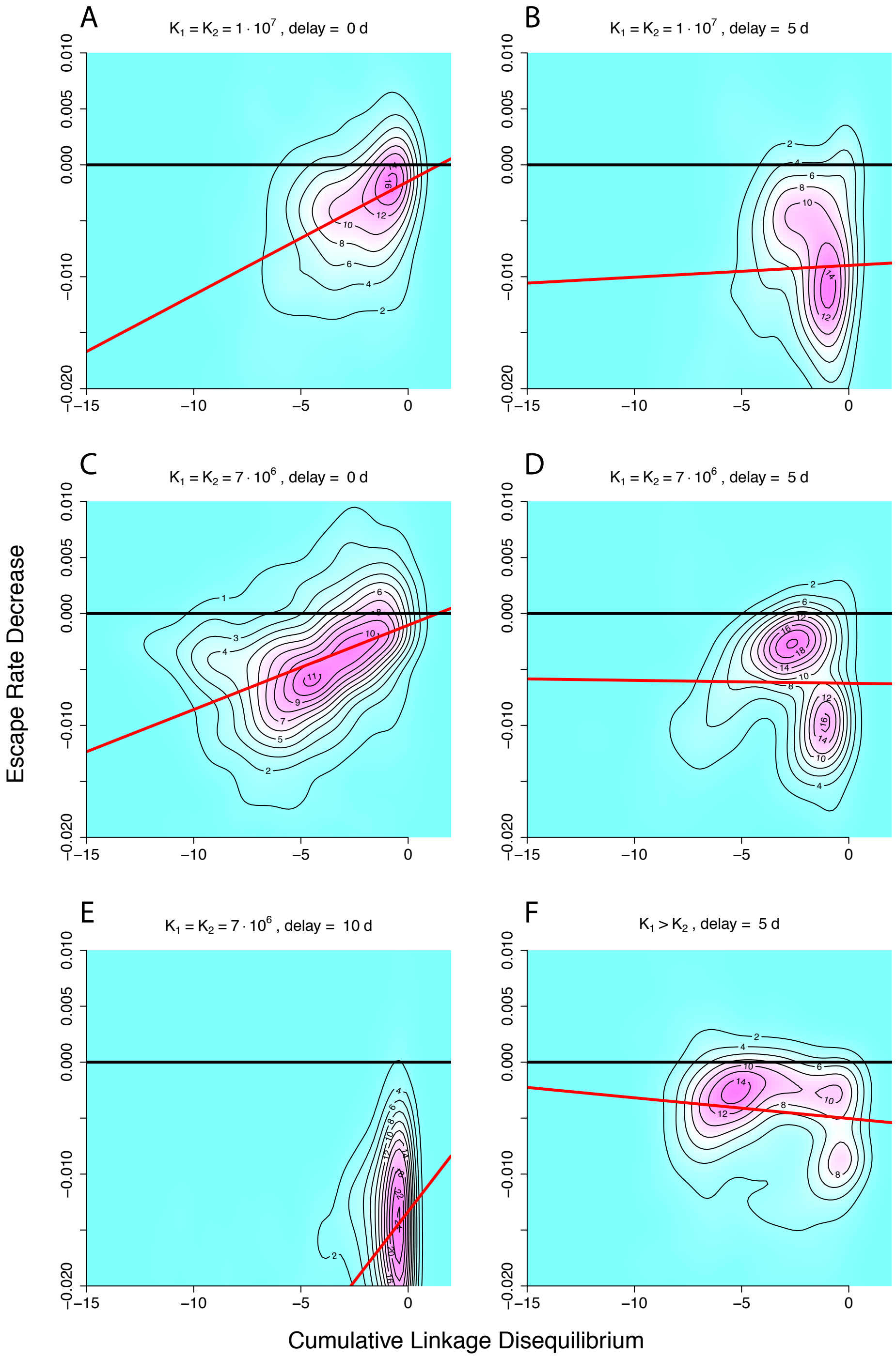

Figure S7. Density plot of six cases of negative cumulative linkage disequilibrium versus escape rate decrease values. Positive cumulative linkage disequilibrium values were ignored. Black line: Base line through the origin. The strength of the $CD8^{+}$ T cell functions as well as the time delays between them are indicated above each plot. An association for equally strong and synchronously elicited immune responses is detected in cases A) and C) (more pronounced for C)). An increase in time delay leads to the appearance of a second density peak at about zero cumulative linkage disequilibrium and negative escape rate decrease values (see cases B), D) and E)). This density peak becomes more pronounced as the time delay between the responses increases from C) over D) to E), as well as from A) to B). Case F) deviates from to the general patterns observed.

REFERENCES

Gerrish P, Lenski R. The fate of competing beneficial mutations in an asexual population. *Genetica* **102** (1998) 127–144.

Desai MM, Fisher DS. Beneficial mutation selection balance and the effect of linkage on positive selection. *Genetics* **176** (2007) 1759–1798.

Tsimring LS, Levine H, Kessler DA. RNA virus evolution via a fitness-space model. *Physical Review Letters* **76** (1996) 4440–4443.

Rouzine IM, Wakeley J, Coffin JM. The solitary wave of asexual evolution. *Proceedings of the National Academy of Sciences* **100** (2003) 587–592.

Rouzine IM, Coffin JM. Evolution of human immunodeficiency virus under selection and weak recombination. *Genetics* **170** (2005) 7–18.

Ganusov VV, Goonetilleke N, Liu MK, Ferrari G, Shaw GM, McMichael AJ, et al. Fitness costs and diversity of the cytotoxic t lymphocyte (ctl) response determine the rate of ctl escape during acute and chronic phases of hiv infection. *Journal of Virology* **85** (2011) 10518–10528. doi:10.1128/JVI.00655-11.

Asquith B, Edwards CT, Lipsitch M, McLean AR. Inefficient cytotoxic T lymphocyte–mediated killing of HIV-1–infected cells in vivo. *PLoS Biology* **4** (2006) e90.

Mansky LM, Temin HM. Lower in vivo mutation rate of human immunodeficiency virus type 1 than that predicted from the fidelity of purified reverse transcriptase. *Journal of Virology* **69** (1995) 5087–5094.

Lalić J, Cuevas JM, Elena SF. Effect of host species on the distribution of mutational fitness effects for an RNA virus. *PLoS genetics* **7** (2011) e1002378.

Sanjuán R, Moya A, Elena SF. The distribution of fitness effects caused by single-nucleotide substitutions in an RNA virus. *Proceedings of the National Academy of Sciences of the United States of America* **101** (2004) 8396–8401.

Mostowy R, Kouyos R, Fouchet D, Bonhoeffer S. The role of recombination for the coevolutionary dynamics of HIV and the immune response. *PloS One* **6** (2011) e16052.

Jung A, Maier R, Vartanian JP, Bocharov G, Jung V, Fischer U, et al. Recombination: Multiply infected spleen cells in HIV patients. *Nature* **418** (2002) 144–144.

Josefsson L, Palmer S, Casazza J, Ambrozak D, Kearney M, Shao W, et al. Analysis of HIV DNA molecules in paired peripheral blood and lymph node tissue samples from chronically infected patients. *Antiviral Therapy* (INT MEDICAL PRESS LTD 2-4 IDOL LANE, LONDON EC3R 5DD, ENGLAND) (2010), vol. 15, A41–A41.

Neher RA, Leitner T. Recombination rate and selection strength in HIV intra-patient evolution. *PLoS Computational Biology* **6** (2010) e1000660.

Batorsky R, Kearney MF, Palmer SE, Maldarelli F, Rouzine IM, Coffin JM. Estimate of effective recombination rate and average selection coefficient for HIV in chronic infection. *Proceedings of the National Academy of Sciences* **108** (2011) 5661–5666.

De Boer R. Understanding the failure of CD8+ T-cell vaccination against simian/human immunodeficiency virus. *Journal of Virology* **81** (2007) 2838–2848.

Hockett RD, Kilby JM, Derdeyn CA, Saag MS, Sillers M, Squires K, et al. Constant mean viral copy number per infected cell in tissues regardless of high, low, or undetectable plasma HIV RNA. *The Journal of Experimental Medicine* **189** (1999) 1545–1554.

Reilly C, Wietgrefe S, Sedgewick G, Haase A. Determination of simian immunodeficiency virus production by infected activated and resting cells. *AIDS* **21** (2007) 163–168.

Chen HY, Di Mascio M, Perelson AS, Ho DD, Zhang L. Determination of virus burst size in vivo using a single-cycle SIV in rhesus macaques. *Proceedings of the National Academy of Sciences* **104** (2007) 19079–19084.

De Boer RJ, Ribeiro RM, Perelson AS. Current estimates for HIV-1 production imply rapid viral clearance in lymphoid tissues. *PLoS Computational Biology* **6** (2010) e1000906.

Ramratnam B, Bonhoeffer S, Binley J, Hurley A, Zhang L, Mittler JE, et al. Rapid production and clearance of HIV-1 and hepatitis C virus assessed by large volume plasma apheresis. *The Lancet* **354** (1999) 1782–1785.

Jetzt AE, Yu H, Klarmann GJ, Ron Y, Preston BD, Dougherty JP. High rate of recombination throughout the human immunodeficiency virus type 1 genome. *Journal of Virology* **74** (2000) 1234–1240.

Zhuang J, Jetzt AE, Sun G, Yu H, Klarmann G, Ron Y, et al. Human immunodeficiency virus type 1 recombination: rate, fidelity, and putative hot spots. *Journal of Virology* **76** (2002) 11273–11282.

Fernandez CS, Stratov I, De Rose R, Walsh K, Dale CJ, Smith MZ, et al. Rapid viral escape at an immunodominant simian-human immunodeficiency virus cytotoxic T-lymphocyte epitope exacts a dramatic fitness cost. *J. Virol.* **79** (2005) 5721–5731.

Henn Mea. Whole genome deep sequencing of HIV-1 reveals the impact of early minor variants upon immune recognition during acute infection. *PLoS Pathogens* **8** (2012) e1002529.

Goonetilleke N, Liu M, Salazar-Gonzalez J, Ferrari G, Giorgi E, Ganusov V, et al. The first T cell response to transmitted/founder virus contributes to the control of acute viremia in HIV-1 infection. *The Journal of Experimental Medicine* **206** (2009) 1253–1272.

Ewens WJ. *Mathematical population genetics: I. Theoretical introduction*, vol. 27 (Springer) (2004).

Jackson CH. Multi-State Models for Panel Data: The msm Package for R. *Journal of Statistical Software* **38** (2011) 1–29.

Team RDC. *R: A Language and Environment for Statistical Computing*. R Foundation for Statistical Computing, Vienna, Austria (2012). ISBN 3-900051-07-0.
